# Supplementary material for: Tailoring grain boundary stability of zinc-titanium alloy for long-lasting aqueous zinc batteries
Source: Nat Commun. 2023 Nov 4;14:7080. doi: 10.1038/s41467-023-42919-7 (PMC10625522; doi:10.1038/s41467-023-42919-7)
Supplement: Supplementary file 1 — Supplementary Information [file 41467_2023_42919_MOESM1_ESM.pdf]

Supplementary Information for

Tailoring grain boundary stability of zinc-titanium alloy for long-lasting aqueous zinc batteries

Yunxiang Zhao[1], Shan Guo[1], Manjing Chen[1], Bingan Lu[2], Xiaotan Zhang[1]*, Shuquan Liang[1]* & Jiang Zhou[1]*

[1]School of Materials Science and Engineering, Hunan Provincial Key Laboratory of Electronic Packaging and Advanced Functional Materials, Central South University, Changsha 410083, Hunan, China.

[2]School of Physics and Electronics, Hunan University, Changsha 410082, Hunan, China.

*Corresponding authors: zhangxiaotan@csu.edu.cn; lsq@csu.edu.cn; zhou_jiang@csu.edu.cn

Supplementary Figures

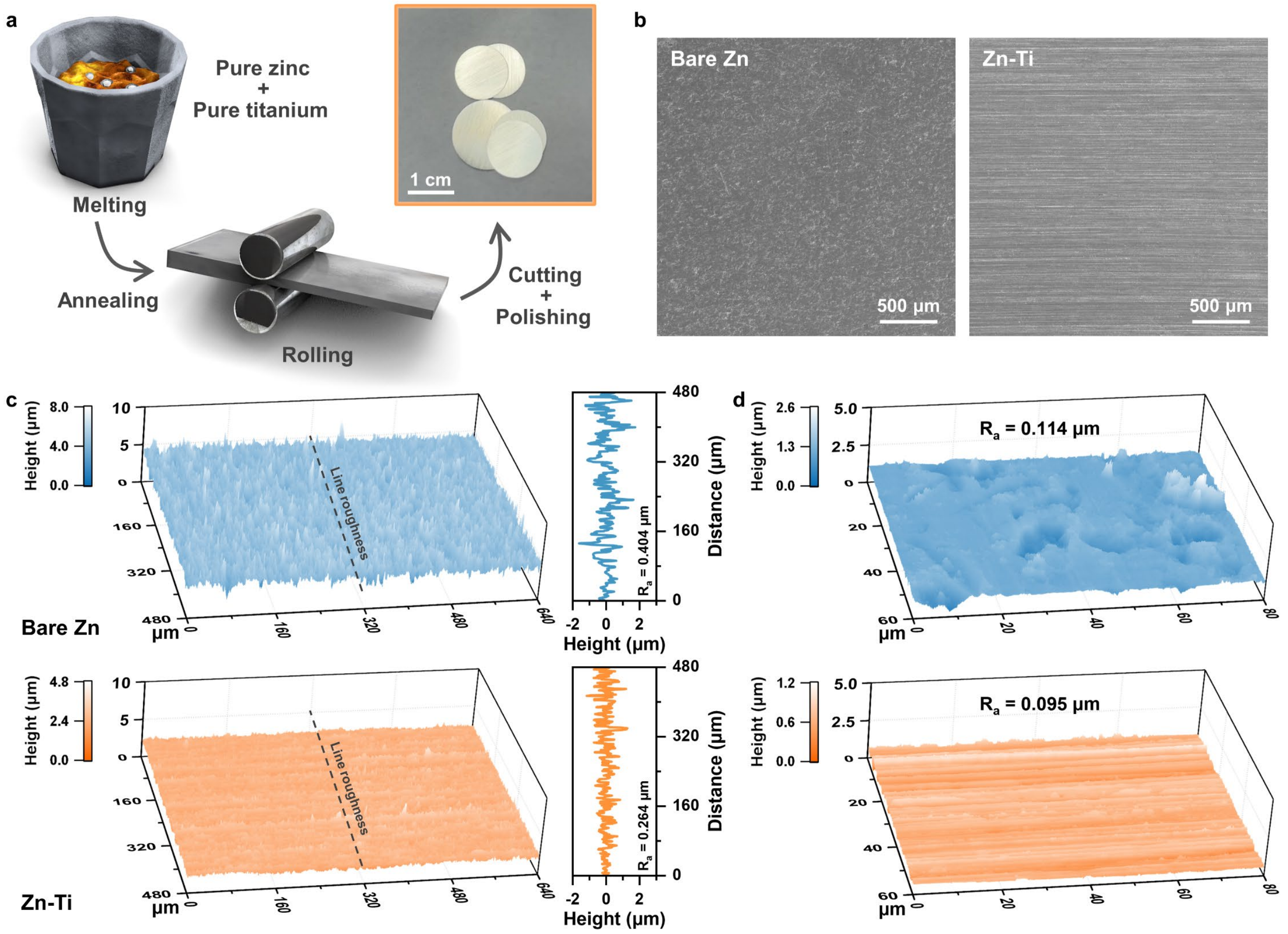

Supplementary Figure 1. Processing and surface morphology characterization of pristine Zn anodes. (a) Fabrication process of Zn-Ti alloy. (b) SEM images of bare Zn and Zn-Ti alloy foils. (c) CLSM imaging of pristine morphologies on bare Zn and Zn-Ti alloy, and corresponding surface profiles extracted along the location marked. (d) CLSM images of bare Zn and Zn-Ti alloy at higher magnification.

The bare Zn surface appears predominantly flat, with minor cracks, while the Zn-Ti alloy foil exhibits regular scratches, resulting from the polishing procedure in the metallurgical process. Nonetheless, the surfaces of both Zn anodes are relatively smooth, characterized by a low surface roughness measuring approximately 0.1 μm.

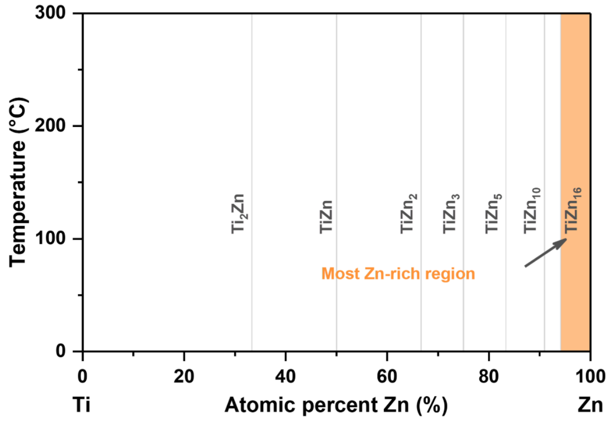

Supplementary Figure 2. Zn-Ti binary phase diagram.

Based on the phase diagram, the utilization ratio of Zn anode can be maximized within the most Zn-rich region. Within this range, to ensure the presence of sufficient Ti-containing IMCs and to avoid dendritic structures, an empirical Zn-Ti weight ratio of 99.5:0.5 was employed. In this scenario, the alloy undergoes sequential phase transitions during the cooling process[1], ultimately crystallizing into a Zn-rich solid solution and $TiZn_{16}$ IMCs.

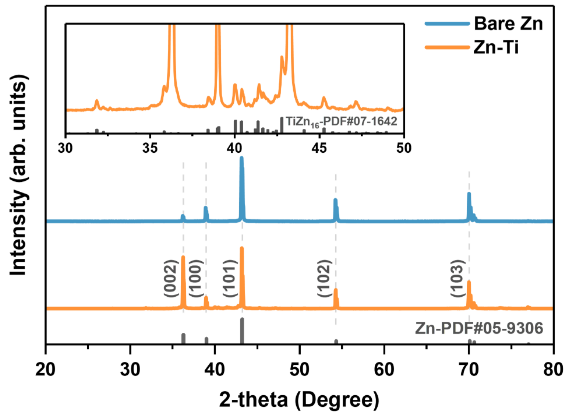

Supplementary Figure 3. XRD patterns of bare Zn and Zn-Ti alloy.

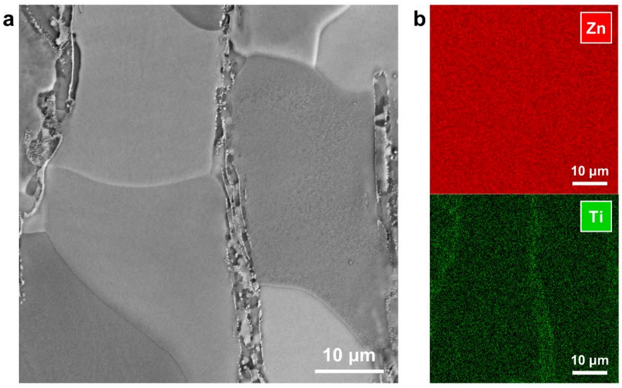

Supplementary Figure 4. Microstructure of Zn-Ti alloy after twin-jet electropolishing. (a) SEM backscattered electron image and corresponding (b) elemental mapping of Zn-Ti alloy.

Following the twin-jet electropolishing procedure in a strongly acidic electrolyte, the partial preservation of IMCs to some extent demonstrates their good corrosion resistance. The reason for not employing mechanical grinding and polishing lies in the inherent lower hardness of Zn alloys, which renders them prone to generating numerous scratches during the process, thus severely impeding the observation of microstructure features.

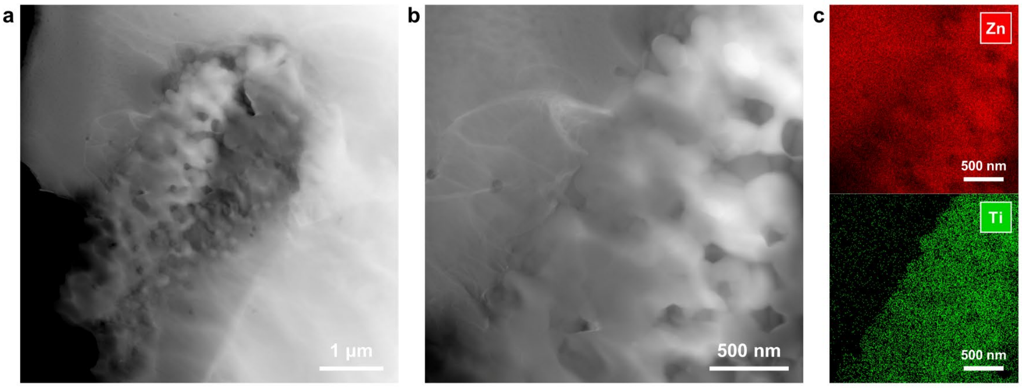

Supplementary Figure 5. Structural characterizations of Zn-Ti alloy after twin-jet electropolishing. (a-b) TEM images and corresponding (c) elemental mapping of Zn and Ti species.

The TEM findings unambiguously confirm the presence of IMCs at the GBs. The corresponding EDS mapping further reveals a stacked distribution of Ti and Zn within the IMCs region, which is ascribed to the $TiZn_{16}$ IMCs.

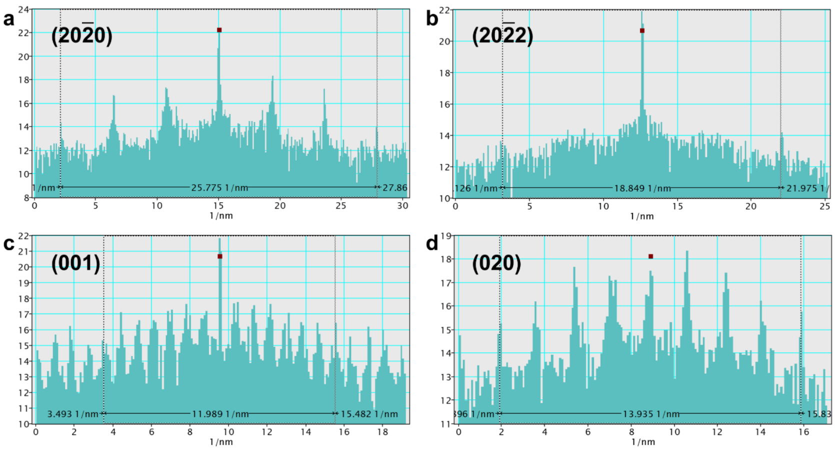

Supplementary Figure 6. The intensity line profiles indicating the interlayer spacings of (a-b) Zn and (c-d) $TiZn_{16}$.

The interlayer spacings of 0.116 nm and 0.106 nm were identified above the interface, matching well with the (20$\bar{2}$0) and (20$\bar{2}$2) planes of Zn. The interlayer spacings of 1.168 nm and 0.574 nm were identified below the interface, matching well with the (001) and (020) planes of $TiZn_{16}$.

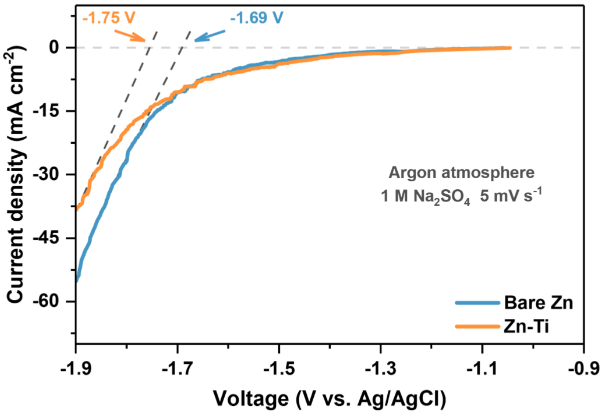

Supplementary Figure 7. LSV curves recorded under argon atmosphere.

Sodium salts and inert gas shielding were employed to exclude the effects of competing Zn deposition process and to eliminate the influence of ambient atmosphere, respectively[2,3].

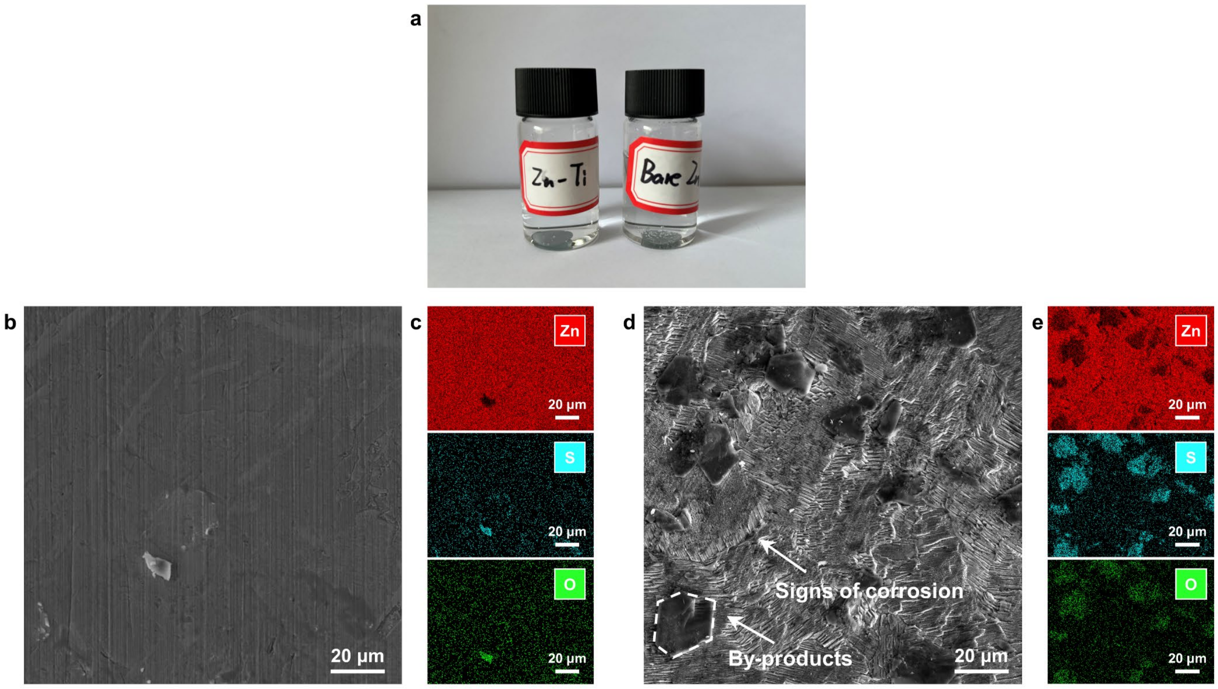

Supplementary Figure 8. Corrosion behavior of Zn anodes under prolonged aging. (a) Optical photograph of the Zn anodes during immersion. SEM and corresponding EDS elemental mapping of (b-c) Zn-Ti alloy and (d-e) bare Zn after 10 days of immersion.

Once the HER occurs, a significant amount of hydroxide ions is generated. The localized alkaline environment affects the chemical surroundings at the interface, thereby prompting the precipitation of zinc hydroxysulfate ($Zn_4SO_4(OH)_6 \cdot xH_2O$) from the electrolyte onto the surface of Zn anodes. The formation process can be represented as:

$$4Zn^{2+} + 6OH^- + {SO_4}^{2-} + xH_2O \rightarrow Zn_4SO_4(OH)_6 \cdot xH_2O \quad (1)$$

It is noteworthy that during the immersion process, the surface of Zn-Ti alloy exhibits only a few sporadic bubbles, in sharp contrast to the evident bubble accumulation observed on bare Zn.

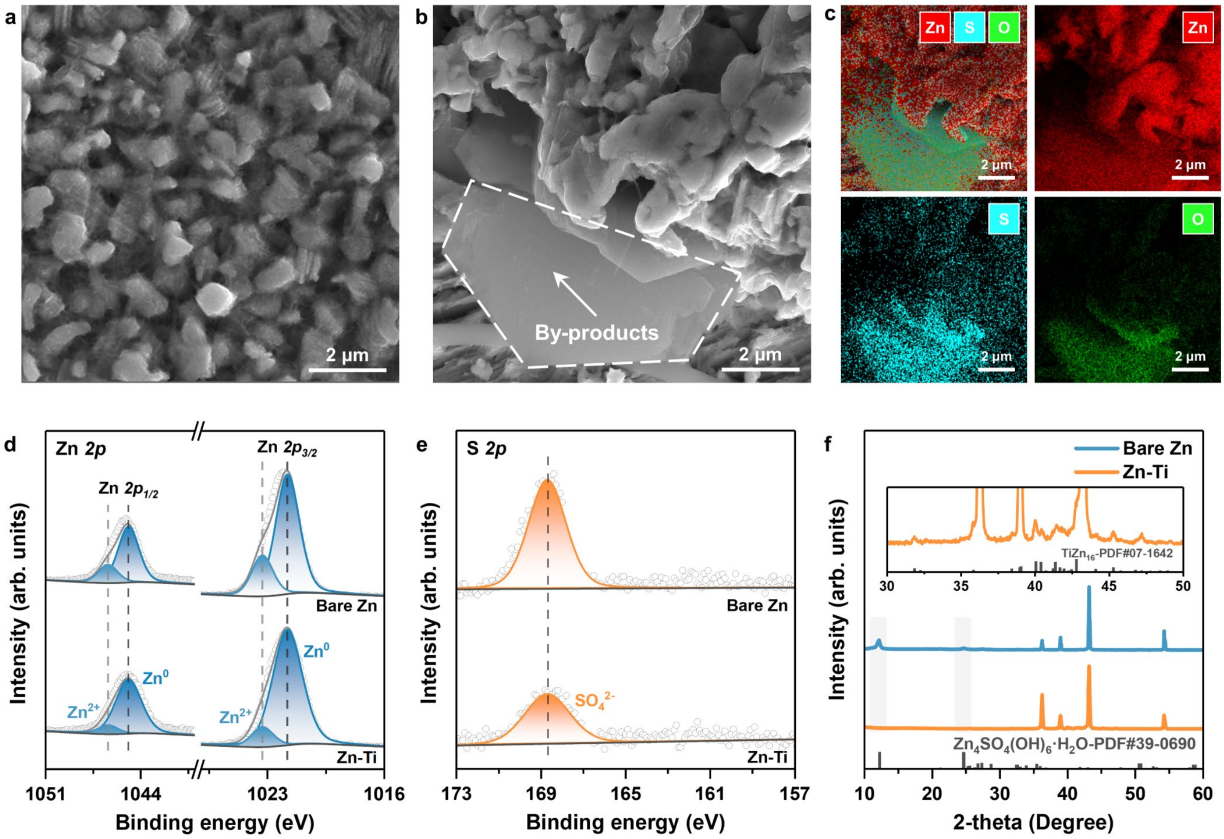

Supplementary Figure 9. Top-view SEM images of (a) Zn-Ti alloy and (b) bare Zn after 50 cycles at 1 mA cm^{-2}/1 mAh cm^{-2}. (c) The EDS elemental mapping of deposits on bare Zn. (d) Zn *2p*, (e) S *2p* XPS spectra and (f) XRD patterns of Zn anodes after cycling.

Considering that the HER primarily occurs during the cathodic process[4], a typical deposition morphology was selected to identify the by-products. Following the completion of cycling in the symmetrical cell, the electrode on deposition side was immediately extracted and rinsed with deionized water to remove any residual electrolyte. The sample was then dried under vacuum and employed for various measurements, including SEM, XRD and X-ray photoelectron spectroscopy (XPS) analysis. Top-view SEM observations reveal the existence of hexagonal flake-like species on the bare Zn surface, and corresponding EDS elemental mapping confirms that the predominant constituents of these species are Zn, S, and O. The application of further XPS analysis unveils the surface chemical composition. The S *2p* XPS spectra indicate the distinct SO_4^{2-} peaks at 168.7 eV on the surface of Zn anodes after cycling[5], with significantly higher intensity observed on bare Zn. Meanwhile, the Zn $2p_{3/2}$ XPS spectra can be deconvoluted into two peaks at 1023.3 eV and 1021.8 eV, corresponding to Zn^{2+} and Zn^0, respectively[6]. Notably, the proportion of Zn^{2+} for bare Zn (23.5%) exceeds that in the Zn-Ti alloy (11.4%). Combined with XRD results, the by-product resulting from parasitic reactions is identified as $Zn_4SO_4(OH)_6·H_2O$ (PDF#39-0690), exhibiting a more pronounced effect observed for bare Zn.

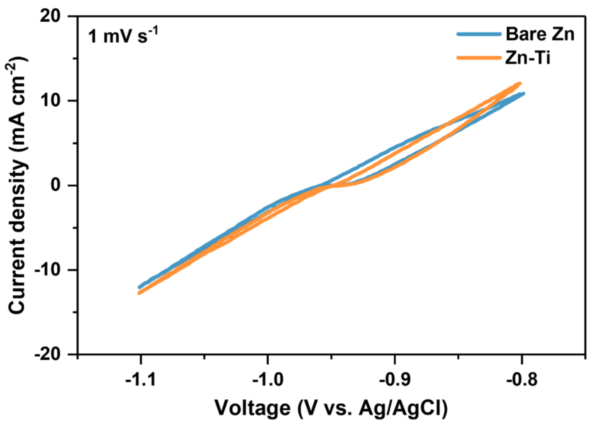

Supplementary Figure 10. CV curves of plating/stripping process in the three-electrode system.

The analogous centrosymmetric loops reflect the similar Zn plating/stripping behavior for the two anodes. Nevertheless, Zn-Ti alloy exhibits a more pronounced current response, indicating its improved reaction kinetics. Moreover, no other peaks can be observed for Zn-Ti alloy, proving that the IMCs can be thermodynamically stabilized at GBs.

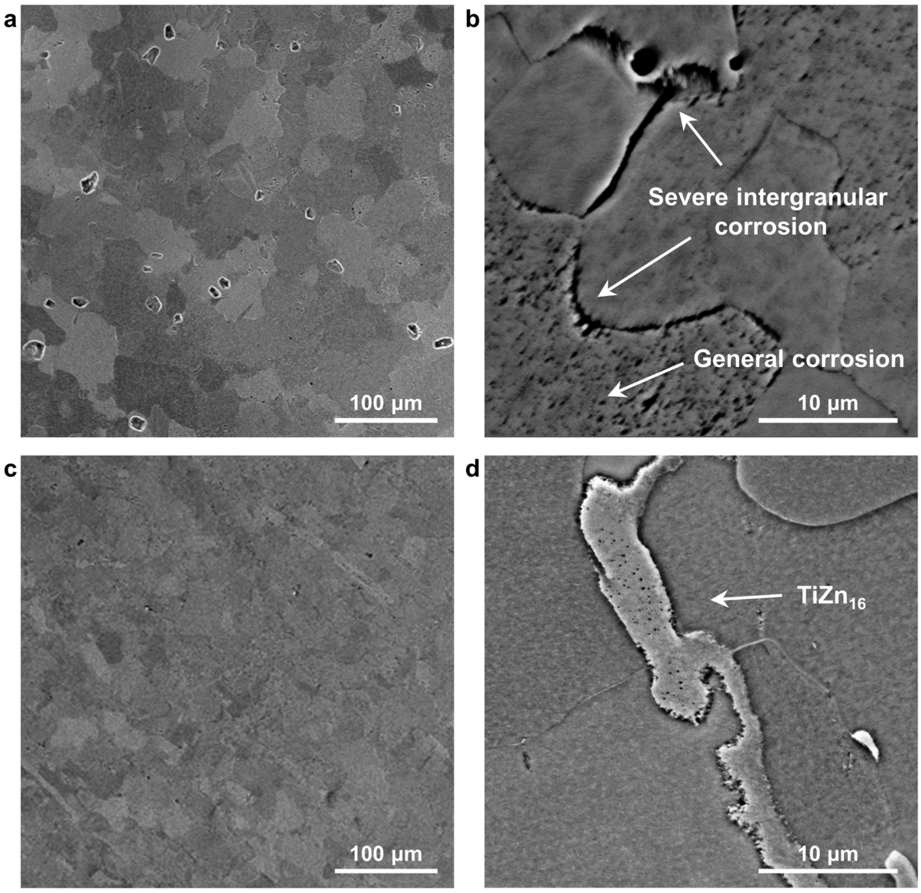

Supplementary Figure 11. SEM images of (a-b) bare Zn and (c-d) Zn-Ti alloy after immersion in 3 M $ZnSO_4$ electrolyte for 24 h.

To accurately identify the corrosion initiation sites and obtain high-quality SEM and EBSD IPF mapping images, corrosion products on the surface of Zn anodes were removed with deionized water. Additional SEM images were employed to differentiate the unrecognized white parts within EBSD images and to provide supplementary information about the corrosion behavior. Specifically, the unrecognized phase, characterized by its continuous distribution along GBs, can be attributed to $TiZn_{16}$ IMCs, while the irregularly shaped ones are identified as corrosion pits. After immersion, the surface of bare Zn was observed to contain a significant number of corrosion pits, with higher resolution image revealing severe intergranular corrosion and some general corrosion. On the contrary, Zn-Ti alloy demonstrates excellent resistance to intergranular corrosion and, to a certain extent, also inhibits general corrosion, attributed to the good corrosion resistance of Zn-rich solid solution.

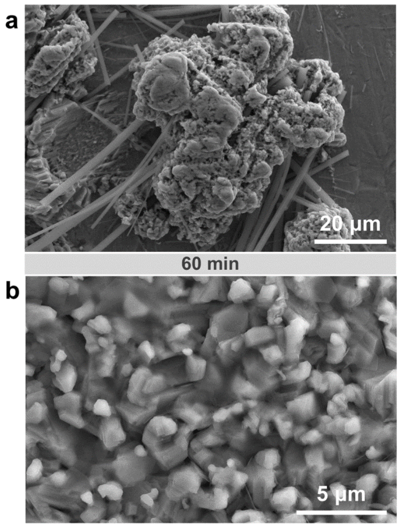

Supplementary Figure 12. SEM images of (a) bare Zn and (b) Zn-Ti alloy after 60 min of plating.

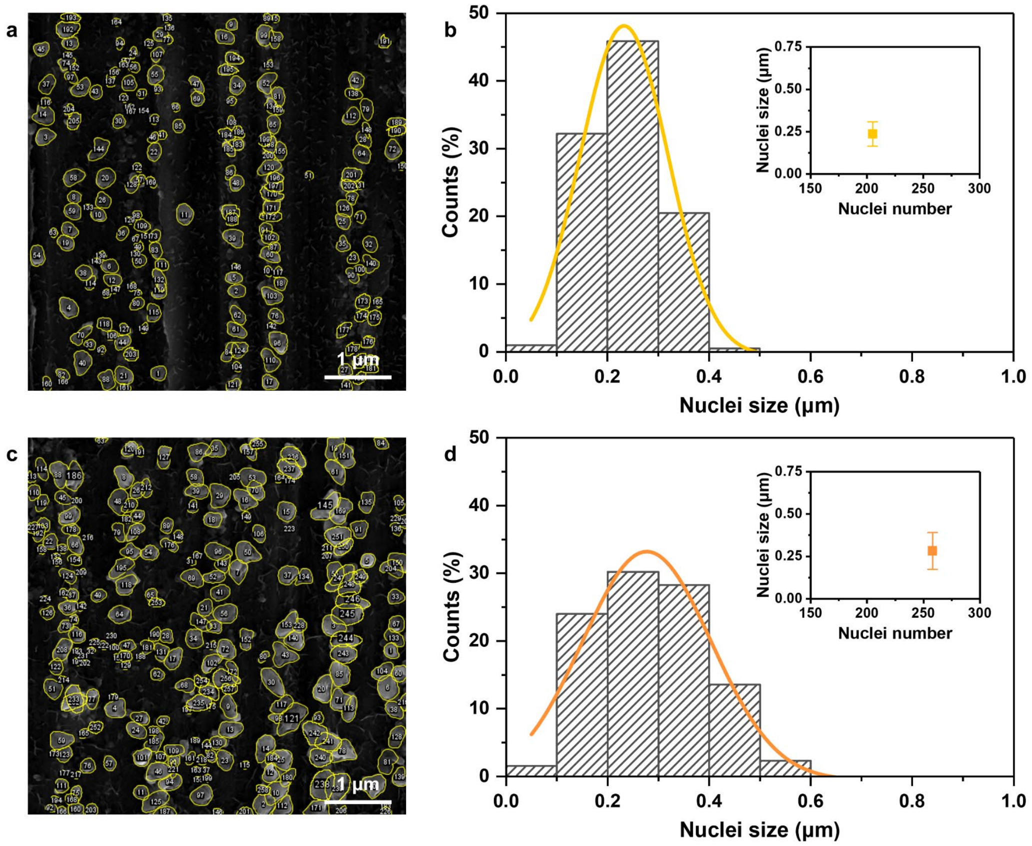

Supplementary Figure 13. SEM images of Zn deposits on Zn-Ti alloy after plating for (a) 15 s and (c) 30 s, along with the corresponding (b, d) histograms illustrating the sizes of nuclei (insets display the statistical nuclei number and average nuclei size). The error bars in the insets represent the standard deviation.

The evolution of nuclei size and number during the nucleation process was quantitatively investigated using StarDist, a publicly available plugin for Fiji (Fiji is just ImageJ). The histograms of the statistical data reveal an increase in the average size of Zn nuclei over time, accompanied by a slightly more scattered distribution, due to the generation of new Zn nuclei.

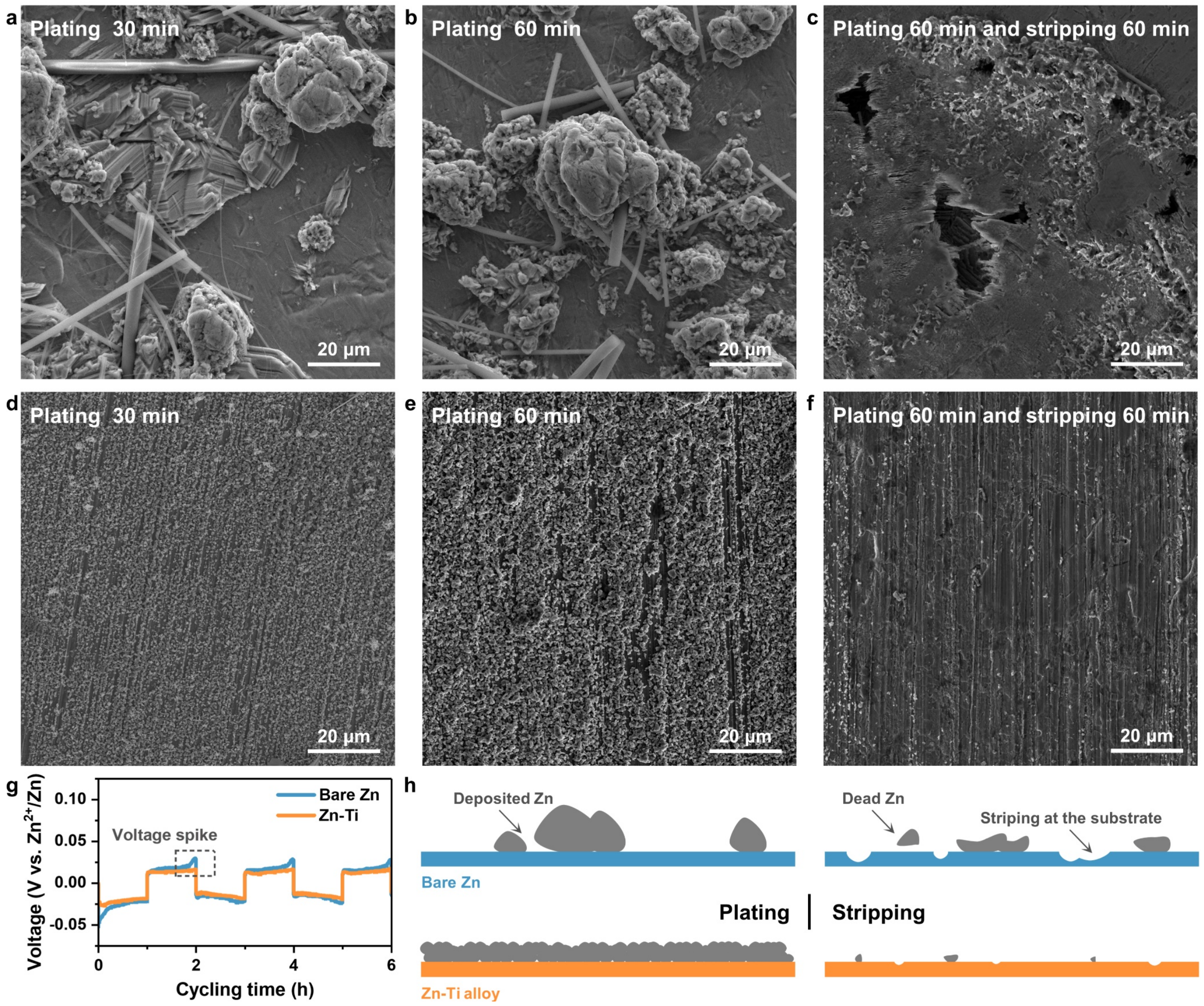

Supplementary Figure 14. SEM images of (a-c) bare Zn and (d-f) Zn-Ti after 30 min of plating, 60 min of plating, and 60 min of plating followed by 60 min of stripping. (g) Voltage profiles of symmetrical cells at 1 mA cm^{-2}/1 mAh cm^{-2}. (h) Schematic diagram of Zn plating and stripping on bare Zn and Zn-Ti alloy.

The size of Zn deposits on bare Zn and Zn-Ti alloy during the plating process exhibits a significant difference. To facilitate a better comparison, distinct scale bars have been utilized in the manuscript. The voltage profiles observed during the subsequent stripping process can be divided into two parts: the stable voltage stage and the increasing voltage stage. The initial stable voltage corresponds to the stripping of pre-deposited Zn from the former process, which is easy to proceed[7]. Once the strippable pre-deposited Zn is depleted, the stripping process shifts to the substrate, resulting in a voltage spike[8]. Clearly, bare Zn exhibits a short stable voltage stage during this process, followed by a noticeable voltage spike. This indicates the incomplete stripping of pre-deposited Zn (also known as "dead Zn" formation) and the consumption of substrate, as further confirmed by the SEM images. In contrast, the surface of Zn-Ti alloy appears relatively smooth after stripping, highlighting the benefits of Zn-Ti alloy not only for plating but also for the stripping process.

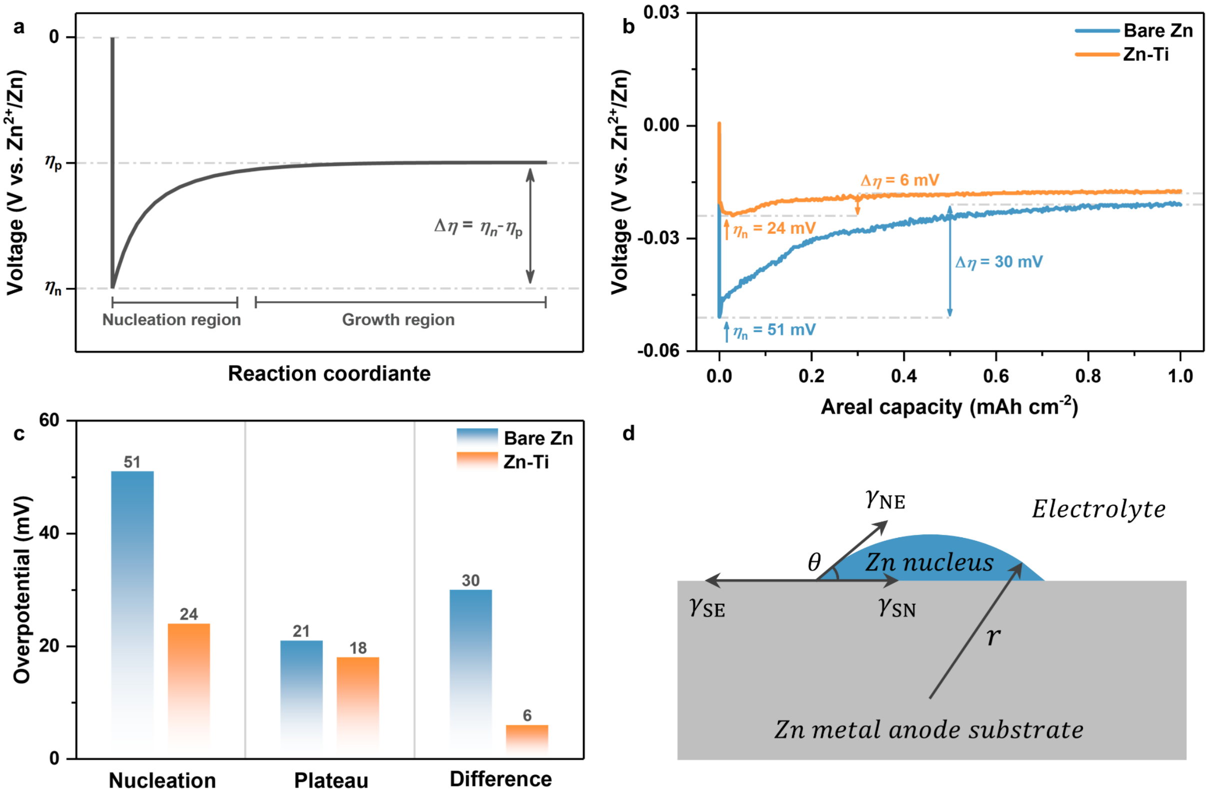

Supplementary Figure 15. Fundamentals of Zn nucleation and growth. (a) Typical voltage profiles of galvanostatic Zn deposition. (b) Voltage profiles of symmetrical cells at 1 mA cm^{-2}/1 mAh cm^{-2}. (c) The calculated overpotential value. (d) Schematic diagram of Zn heterogeneous nucleation on the substrate based on the classical nucleation theory.

Typically, in the case of Zn plating at a constant current density, the voltage experiences a swift decline to a value below 0 V (vs. Zn^{2+}/Zn) and then levels off with a higher value. The nucleation overpotential (η_n) is defined as the onset voltage dip, which is related to the thermodynamic cost of forming a critical atomic cluster, while the mass-transfer controlled plateau overpotential (η_p) describes the growth process[9]. Note that the plateau overpotential is generally lower than the nucleation overpotential, as the addition of Zn atoms to existing nuclei is more favorable than forming a stable atomic cluster[10]. In addition, the overpotential difference ($\Delta\eta$) is defined as the gap between nucleation overpotential and plateau overpotential.

The nucleation of Zn nuclei involves a highly intricate process. From a thermodynamic perspective, the stability of a Zn nucleus deposited on the electrically charged substrate is determined by the combination of bulk free energy, comprising both chemical and electrical, and the interfacial free energy arising from the generation of new interfaces, which can be expressed by[11]:

$$\Delta G_{\mathrm{sys}} = \left(\Delta G_{\mathrm{V}} + \frac{zF\eta}{V_m}\right) S_{\mathrm{V}} r^3 + (\gamma_{\mathrm{NE}} S_{\mathrm{NE}} + \gamma_{\mathrm{SN}} S_{\mathrm{SN}} - \gamma_{\mathrm{SE}} S_{\mathrm{SN}}) r^2 \quad (2)$$

$$S_{\mathrm{V}} = \frac{\pi}{3}(2 - 3\cos\theta + \cos^3\theta) \tag{3}$$

$$S_{\mathrm{NE}} = 2\pi(1 - \cos\theta) \tag{4}$$

$$S_{\mathrm{SN}} = \pi(1 - \cos^2\theta) \tag{5}$$

where ΔG_{V} stands for the volume free energy change (from liquid phase to solid phase), z the number of electrons transferred, F the Faraday's constant, η the overpotential, V_m the molar volume of Zn, S_{V} the volume factor of spherical cap, S_{NE} the curved surface area factor, S_{SN} the bottom surface area factor, γ_{NE} the nucleus/electrolyte interfacial free energy, γ_{SN} the substrate/nucleus interfacial free energy, γ_{SE} the substrate/electrolyte interfacial free energy, θ the contact angle, and r the radius of curvature of the nucleus. By incorporating the quantitative relationship between interfacial tension and contact angle, known as the Young's equation:

$$\gamma_{\mathrm{SN}} - \gamma_{\mathrm{SE}} = -\gamma_{\mathrm{NE}}\cos\theta \tag{6}$$

Equation (2) can be expressed as follows:

$$\Delta G_{\mathrm{sys}} = \left(\Delta G_{\mathrm{V}} + \frac{zF\eta}{V_m}\right) S_{\mathrm{V}} r^3 + \gamma_{\mathrm{NE}}(S_{\mathrm{NE}} - S_{\mathrm{SN}}\cos\theta) r^2 \tag{7}$$

Physically, nucleation is thermodynamically favored when the volumetric and interfacial tension contributions to the energetics of system overcome the critical free energy. Given $d\Delta G_{\mathrm{sys}}/dr = 0$, the critical radius required to form a thermodynamically stable atomic cluster can be derived as:

$$r_{\mathrm{crit}} = \frac{-2\gamma_{\mathrm{NE}} V_m}{zF\eta + \Delta G_{\mathrm{V}} V_m} \tag{8}$$

This indicates that, for the same volume free energy change, the driving force for nucleation is dominated by the bulk electrostatic energy. Here, η_0 is defined as the characteristic overpotential:

$$\eta_0 = \frac{\Delta G_{\mathrm{V}} V_m}{zF} \tag{9}$$

and therefore, the dimensionless overpotential can be written as:

$$\hat{\eta} = \frac{\eta}{\eta_0} \tag{10}$$

By substituting Equation (8) into Equation (7), the critical free energy to heterogeneously form a nucleus is given by:

$$\Delta G_{\mathrm{crit}} = \frac{16\pi\gamma_{\mathrm{NE}}^3}{3\Delta G_{\mathrm{V}}^2}\frac{(2 - 3\cos\theta + \cos^3\theta)}{4(1 + \hat{\eta})^2} \tag{11}$$

and expressed in dimensionless form:

$$\Delta\hat{G}_{\mathrm{crit}} = \frac{\Delta G_{\mathrm{crit}}}{\Delta G_0} = \frac{(2 - 3\cos\theta + \cos^3\theta)}{4(1 + \hat{\eta})^2} \tag{12}$$

where ΔG_0 is the characteristic Gibbs free energy of the system, which also corresponds to the

critical Gibbs free energy for homogeneous nucleation in the electrolyte (θ = 180°) at η = 0. Calculations demonstrate that, for the system with a small contact angle (internal cause), namely zincophilic substrate, the critical free energy is reduced and nucleation occurs at the overpotential of smaller magnitude. On the other hand, the formation of nuclei during electrocrystallization requires the consumption of electrical energy, which is proportional to the applied overpotential (external cause). When a constant current is applied, the cathodic current is directed towards surface charging, and nucleation only occurs once the polarization reaches a certain threshold. Consequently, the higher the required overpotential, the larger the energy barrier, which is energetically unfavorable.

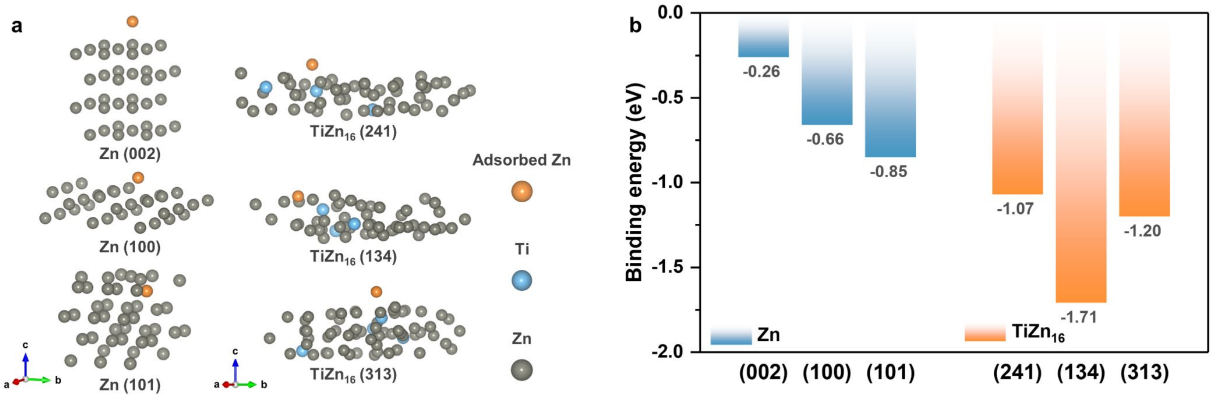

Supplementary Figure 16. Adsorption behavior study of a Zn atom on Zn and $TiZn_{16}$ substrates. (a) Optimal models of Zn atom adsorption on Zn (002) facet, Zn (100) facet, Zn (101) facet, $TiZn_{16}$ (241) facet, $TiZn_{16}$ (134) facet and $TiZn_{16}$ (313) facet. Zn and Ti atoms are colored in gray and blue, respectively, while the adsorbed Zn atom is highlighted in orange. (b) Calculated binding energies of Zn atom with different crystal facets.

The selection of crystal facets is based on the three most prominent diffraction peaks identified in the XRD results.

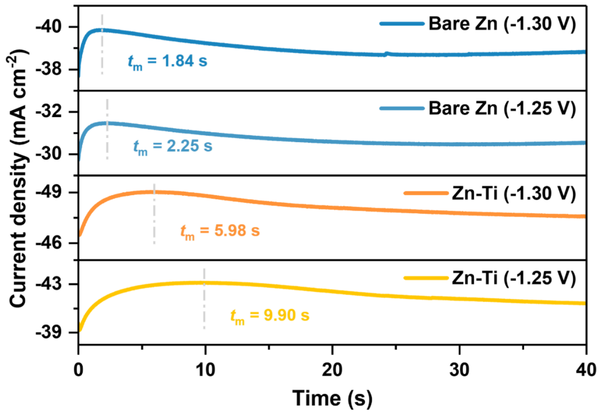

Supplementary Figure 17. Current-time transients obtained at predetermined potentials. j_{m}: peak current, t_{m}: time needed to achieve the peak current.

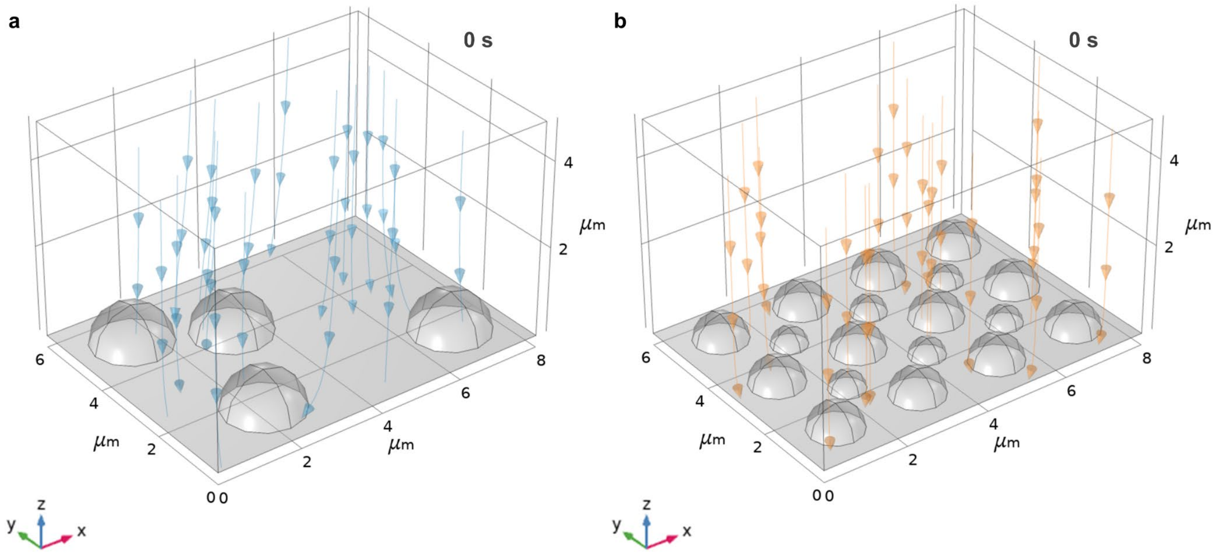

Supplementary Figure 18. Numerical simulation results of the electrolyte current density vectors on (a) bare Zn and (b) Zn-Ti alloy.

The direction of the electrolyte current density vector coincides with the direction of the electric field, which also corresponds to the direction of Zn^{2+} flux.

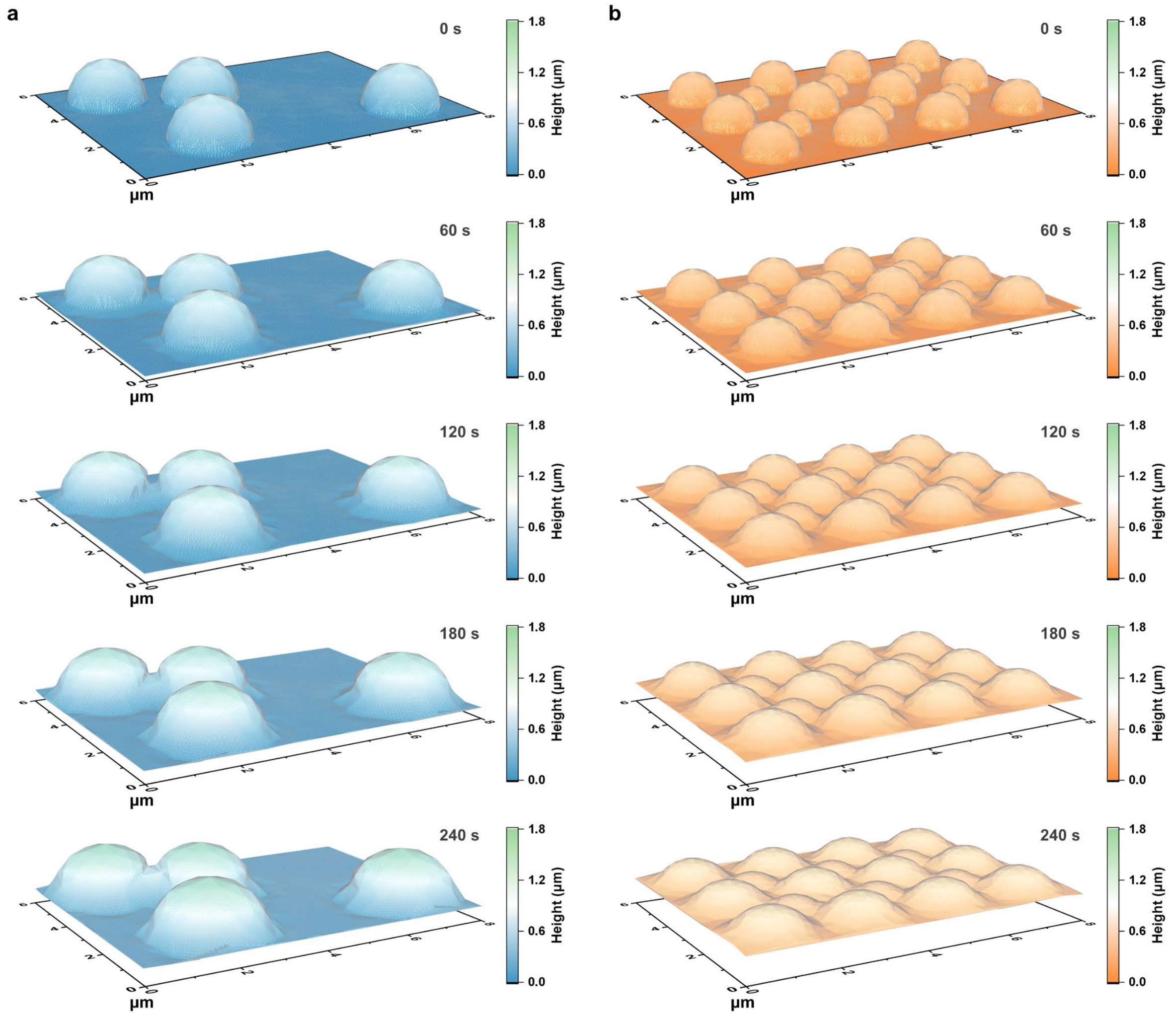

Supplementary Figure 19. The morphological evolution of Zn deposition on (a) bare Zn and (b) Zn-Ti alloy obtained by COMSOL simulations.

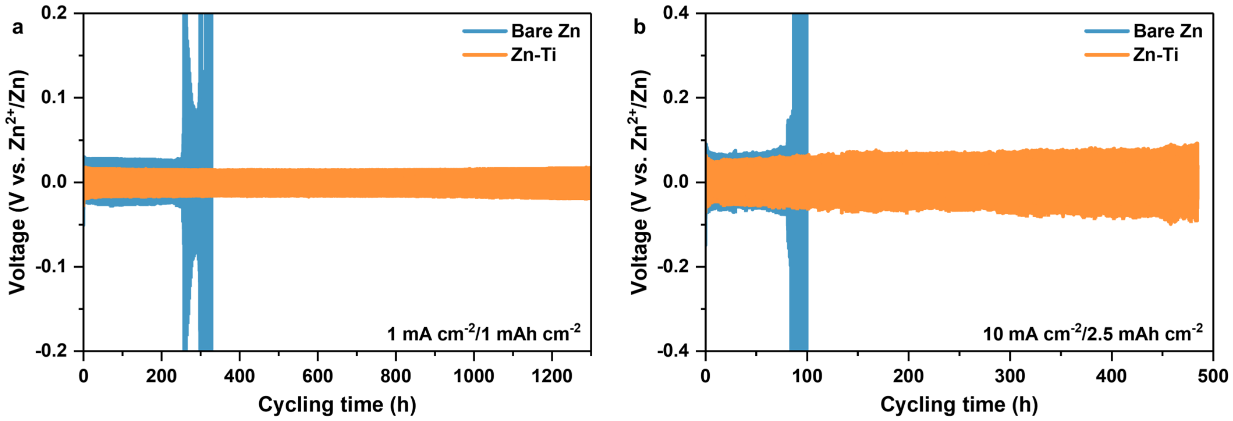

Supplementary Figure 20. Voltage profiles of symmetric cells at (a) 1 mA cm^{-2}/1 mAh cm^{-2} and (b) 10 mA cm^{-2}/2.5 mAh cm^{-2}.

Even at a high current density of 10 mA cm^{-2}, a similar trend is readily achieved for the Zn-Ti alloy.

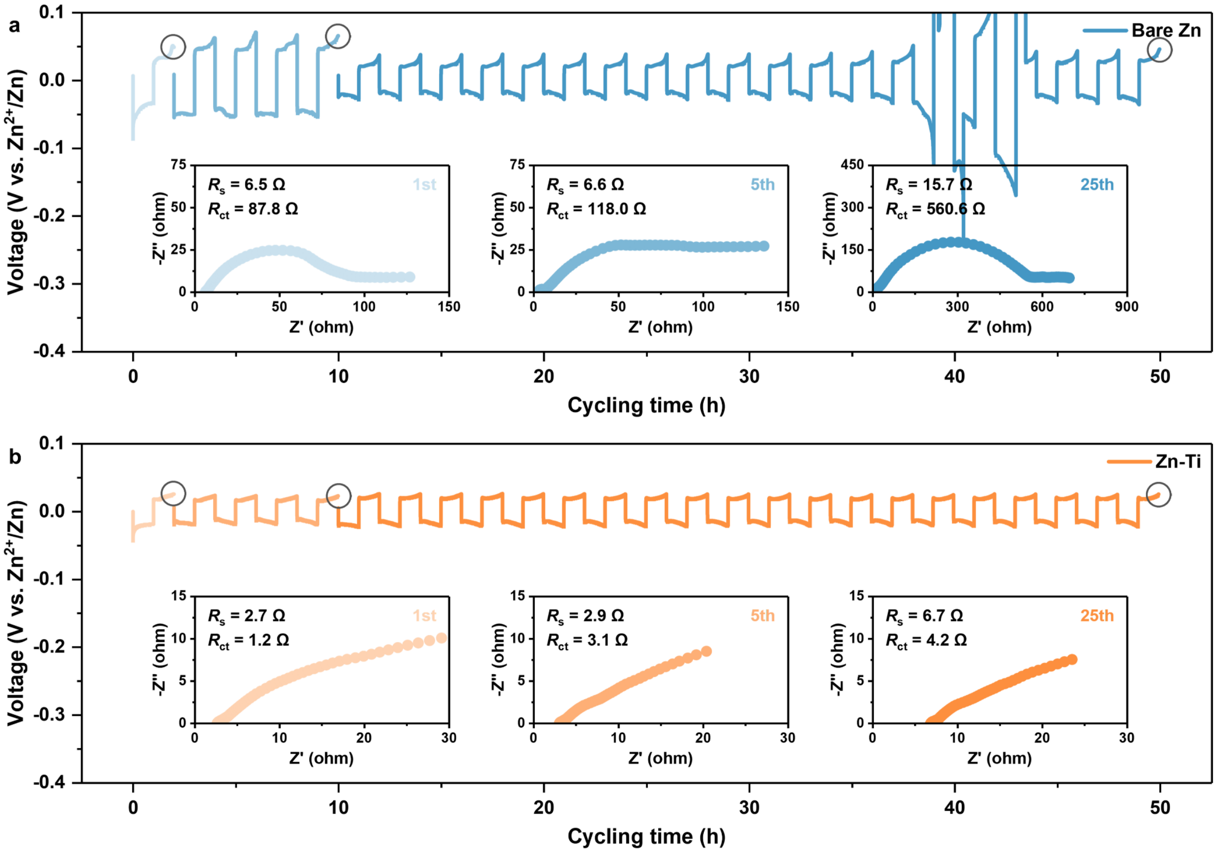

Supplementary Figure 21. Time-dependent Nyquist plots showing the evolution of impedance for (a) bare Zn and (b) Zn-Ti symmetric cells at the current density of 2 mA cm^{-2}.

The charge transfer resistance (R_{ct}) of symmetric cell incorporating with bare Zn increased from 87.8 Ω to 560.6 Ω from 1st to 25th cycle, whereas that of the Zn-Ti alloy stayed almost constant.

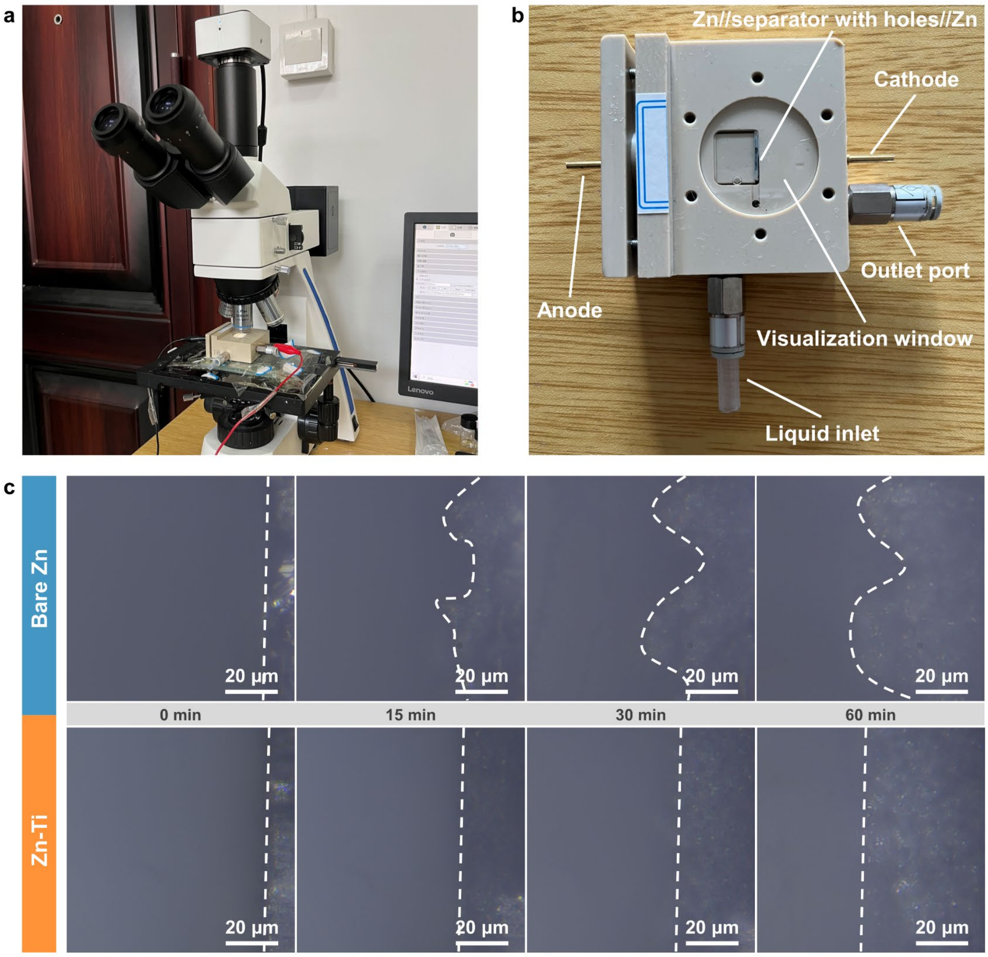

Supplementary Figure 22. The optical photographs of (a) optical microscope and (b) custom-built visualization cell. (c) Operando optical microscope visualization of Zn deposition at 2 mA cm^{-2}.

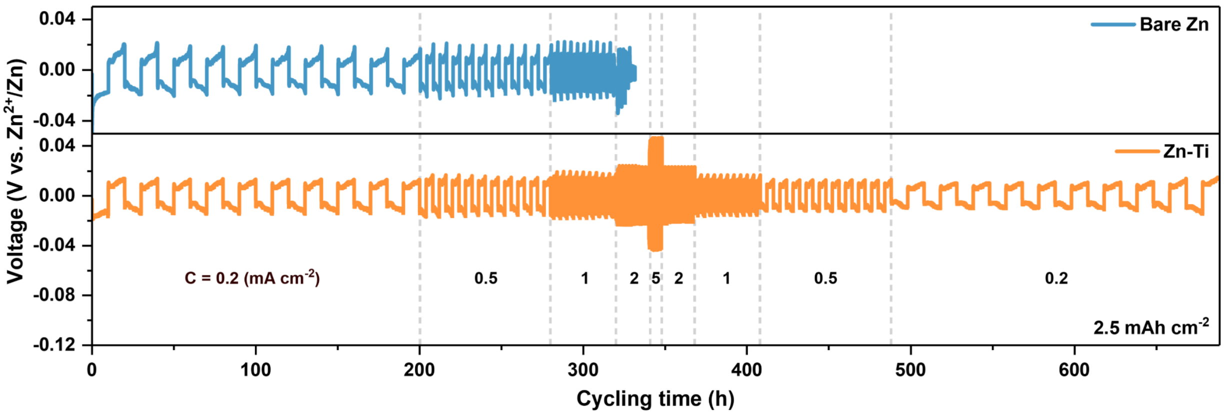

Supplementary Figure 23. Voltage profiles of symmetric cells at various current densities with a fixed areal capacity of 2.5 mAh cm^{-2}.

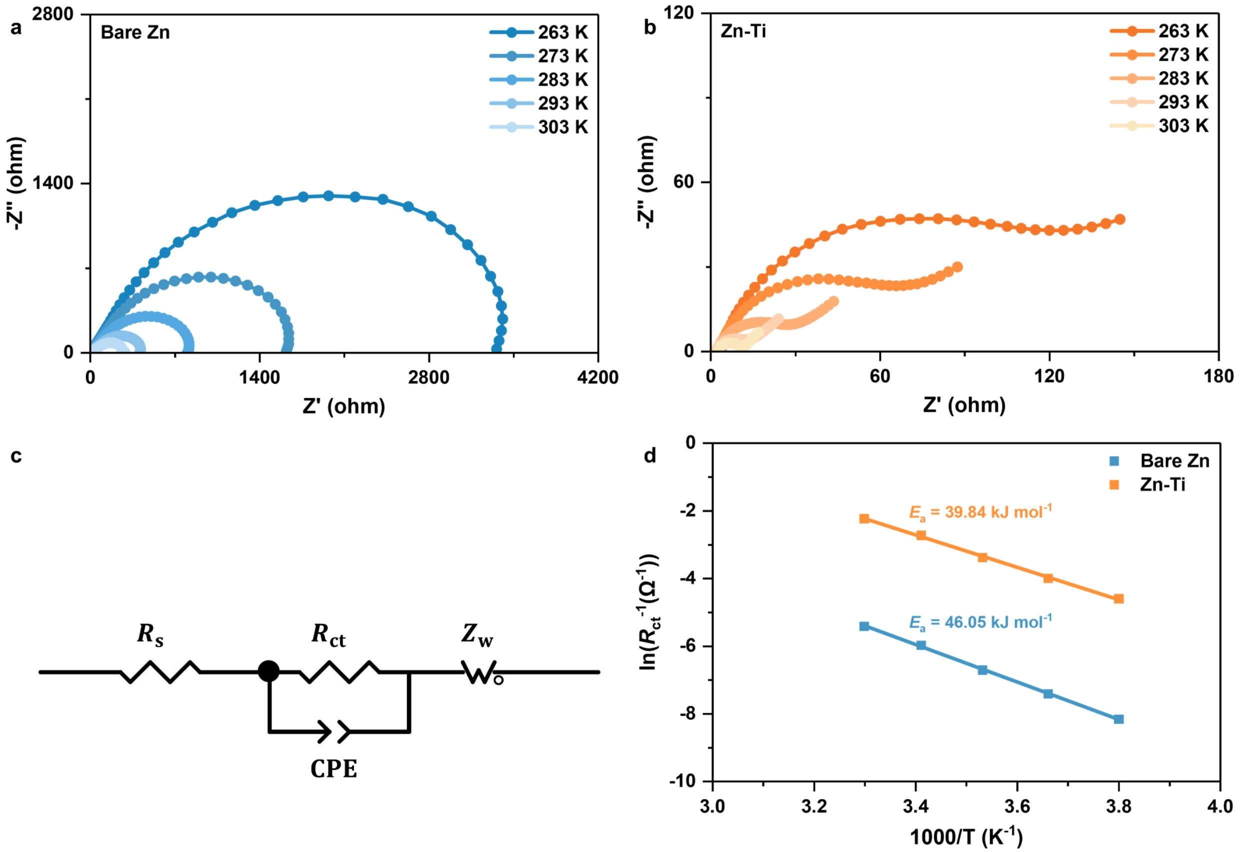

Supplementary Figure 24. Nyquist plots of symmetric cells using (a) bare Zn and (b) Zn-Ti alloy at different temperatures. (c) Typical equivalent circuit model. (d) The fitted curves and the corresponding activation energies calculated by Arrhenius equation.

The Nyquist plot exhibits a characteristic pattern featuring a semicircle in the high-frequency region and a slanted line in the low-frequency region. Randle's equivalent circuit is employed to fit the Nyquist plots, where R_s denotes the ohmic resistance, R_{ct} the charge transfer resistance, CPE the constant phase element, and Z_w the Warburg resistance. Notably, the Zn-Ti alloy demonstrates a more pronounced Warburg behavior, indicating faster bulk diffusion within the alloy structure[12,13]. The charge transfer process at the interface primarily involves desolvation, adsorption and electron transfer[14]. Given the excellent electrical conductivity of metallic electrode, the electron transfer barrier can be considered negligible. Particularly, the desolvation of Zn^{2+} is widely recognized as the rate-determining step for Zn deposition, dictating the reaction kinetics throughout the process[3]. The activation energy for desolvation was deduced through linear fitting, employing Arrhenius equation[15]:

$$\frac{1}{R_{ct}} = Aexp\left(\frac{-E_a}{RT}\right) \quad (13)$$

where R_{ct} represents the charge transfer resistance, A the pre-exponential factor, E_a the activation energy, R the gas constant, and T the temperature in Kelvin. The R_{ct} values at each temperature were estimated by extrapolating the corresponding semicircles in the high-frequency regions.

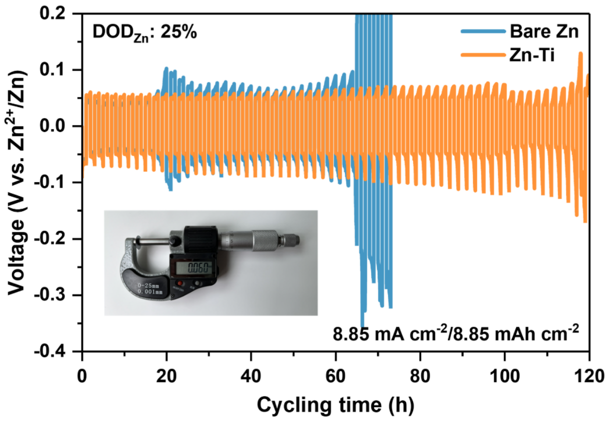

Supplementary Figure 25. Voltage profiles of symmetric cells at 8.85 mA cm^{-2}/8.85 mAh cm^{-2} (inset is an optical photograph of thickness measurement using a screw micrometer).

The Zn foil was first sanded down to a thickness of 60 μm with fine sandpapers, a thickness corresponding to a theoretical capacity of 40 mAh. The depth of discharge ($\mathrm{DOD_{Zn}}$) is identified by:

$$\mathrm{DOD_{Zn}} = \frac{It}{mM} \times 100\% \tag{14}$$

where I refers the applied current, t the galvanostatic time, m the total mass of Zn foil, and M the theoretical capacity of metallic Zn.

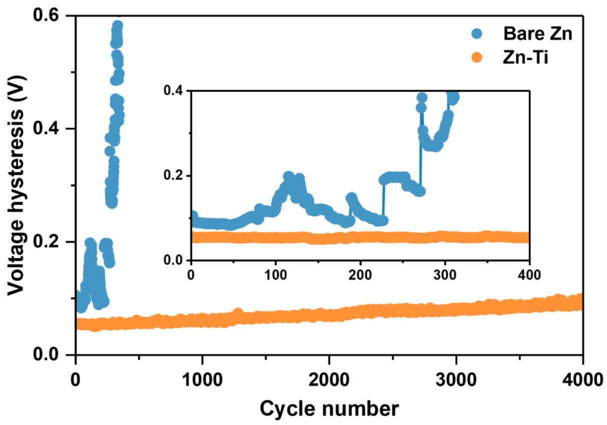

Supplementary Figure 26. The voltage hysteresis of asymmetric cells at 5 mA cm^{-2}/1 mAh cm^{-2}.

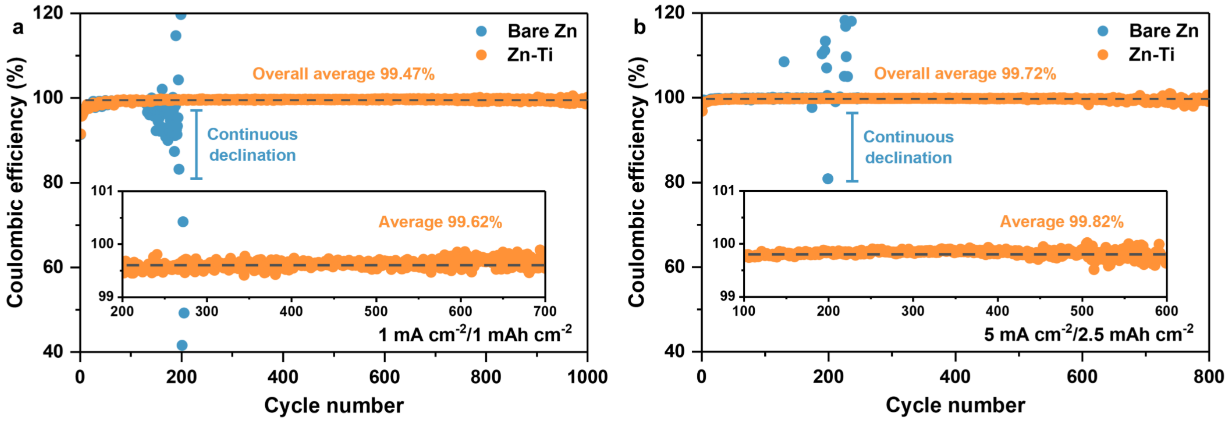

Supplementary Figure 27. The plating/stripping CE of asymmetric cells at (a) 1 mA cm^{-2}/1 mAh cm^{-2} and (b) 5 mA cm^{-2}/2.5 mAh cm^{-2}.

Note that CE increases with increasing current density for identical areal capacity (Fig. 4c and Supplementary Fig. 27a). This is because the Zn plating/stripping CE is correlated with[16]:

$$\frac{\text{Chare capacity from Zn plating/stripping}}{\text{Charge capacity from Zn plating/stripping} + \text{Capacity from HER}} \quad (15)$$

Since HER is time-dependent, a higher current density leads to a shorter time and thus a higher apparent CE. That is, CE is more sensitive to testing time rather than cycle number.

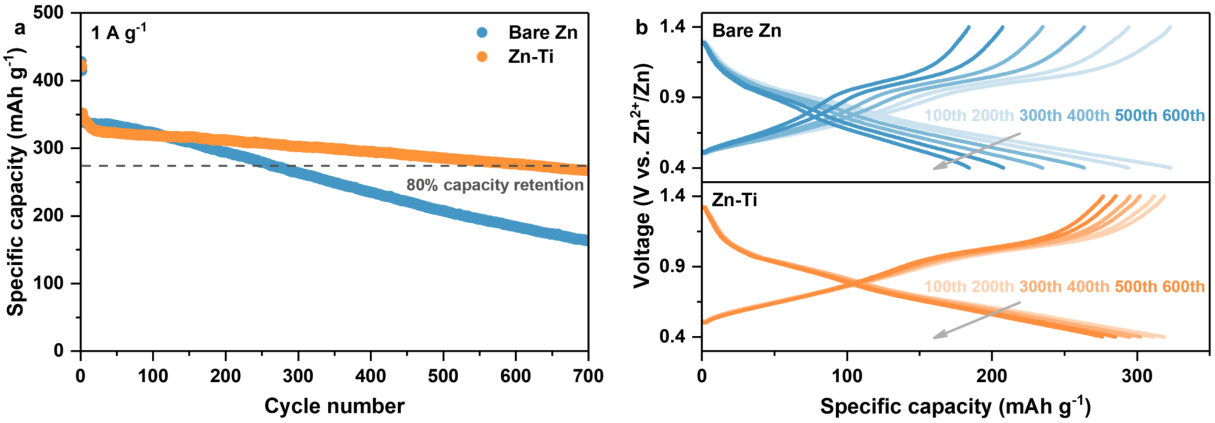

Supplementary Figure 28. Electrochemical performance of the full cells at low current density. (a) Cycling performance and corresponding (b) voltage profiles of full cells at 1 A g^{-1}.

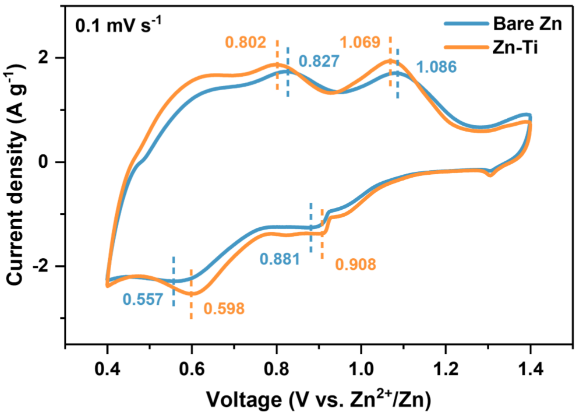

Supplementary Figure 29. CV curves of full cells at a scan rate of 0.1 mV s^{-1}.

The anodic (oxidation) peaks (0.802 V, 1.069 V) of Zn-Ti//$NH_4V_4O_{10}$ are lower than those of bare Zn//$NH_4V_4O_{10}$ (0.827 V, 1.086 V), and the cathodic (reduction) peaks of Zn-Ti//$NH_4V_4O_{10}$ (0.598 V, 0.908 V) are all higher than those of bare Zn//$NH_4V_4O_{10}$ (0.557 V, 0.881 V). These findings suggest a narrower voltage gap for Zn-Ti//$NH_4V_4O_{10}$, consistent with the smaller voltage hysteresis observed in half cells.

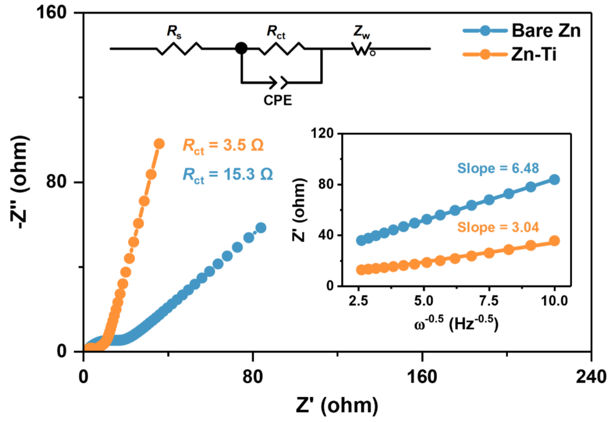

Supplementary Figure 30. Fitted Nyquist plots of full cells after 10 cycles.

In the case of the full cell incorporating Zn-Ti alloy, the steeper slope in the low-frequency region reflects the enhanced Zn^{2+} diffusion kinetics and the lower R_{ct} in the high-frequency region indicates the faster charge transfer.

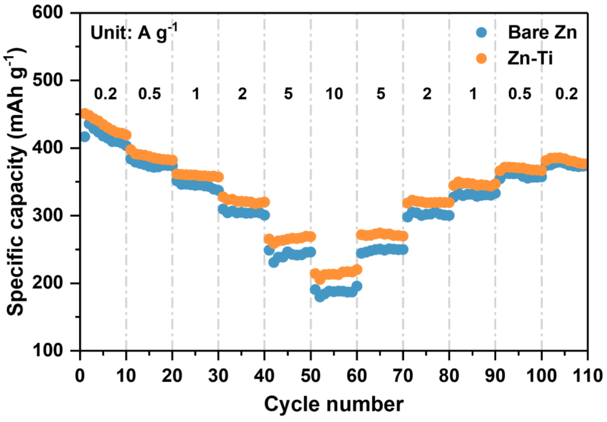

Supplementary Figure 31. Rate performance of full cells from 0.2 to 10 A g^{-1}.

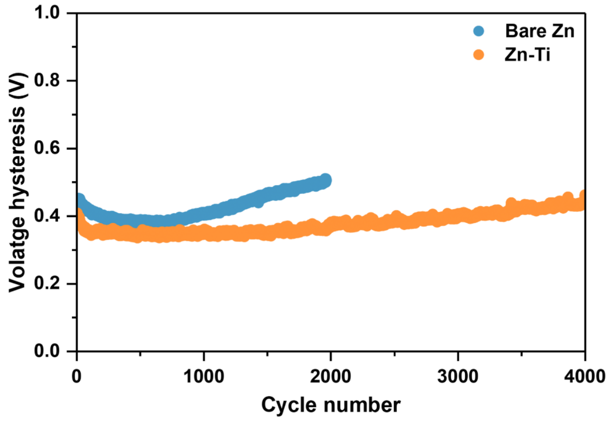

Supplementary Figure 32. Voltage hysteresis of full cells at 5 A g^{-1}.

The decrease in voltage hysteresis during the initial stage can be attributed to the activation processes of full cells. In fact, the slowly evolving voltage hysteresis is closely related to the voltage profiles of half cells[12].

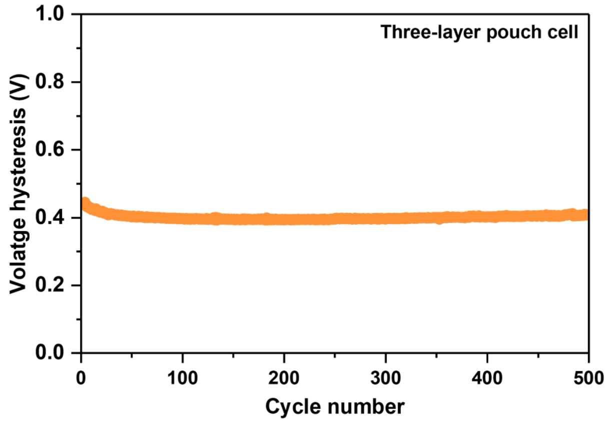

Supplementary Figure 33. Voltage hysteresis of the three-layer pouch cell at 1 A g^{-1}.

Supplementary Tables

Supplementary Table 1. The inductively coupled plasma optical emission spectrometer (ICP-OES) data of Zn-Ti alloy foil.

Element	Ti	Zn
Mass fraction (%)	0.50	Bal.
Atomic ratio (%)	0.68	Bal.

Supplementary Table 2. The performance and cycling conditions of asymmetric cells in recent demonstrations compared to our work. Note: inorganic protective layer (IPL), organic protective layer (OPL), organic-inorganic composite protective layer (OIPL) and Zn-based alloy (ZA).

Type	Cell configuration	Current density (mA cm^{-2})	Coulombic efficiency (%)	Cycle number	Cumulative plated capacity (Ah cm^{-2})	Ref.
IPL	NGO@Zn//Cu	5	99.5	250	0.25	[17]
IPL	ZnO-3D@Zn//Cu	2	99.55	300	0.15	[2]
IPL	IAZO//Cu	2	99.6	400	0.4	[18]
IPL	ZP@Zn//Cu	5	99.5	900	0.9	[19]
IPL	ZnF_2@Zn//Cu	1	~ 99.5	1000	1	[20]
IPL	ZSO@Zn//Cu	1	99.95	140	0.42	[21]
OPL	Zn-PA@Zn//Cu	2	99.9	800	0.8	[22]
OPL	ACG-SEI@Zn//Cu	3	~ 99.7	1800	1.8	[23]
OPL	ZCO@Zn//Cu	4	99.7	200	0.8	[24]
OPL	Zn//TPZA@Cu	5	99.05	300	1.5	[25]
OPL	Zn//PZIL@Cu	2	99.65	1000	1	[26]
OPL	Zn//FCOF@Ti	5	98.4	480	0.48	[27]
OIPL	ex-ZrP//Ti	6	99.5	200	0.6	[28]
OIPL	Zn//PDMS/TiO_{2-x}@Ti	10	99.6	700	0.7	[29]
OIPL	Zn//SIP@Cu	5	98.8	1000	1	[30]
OIPL	Zn//Zn-COF@Cu	10	~ 99.3	150	1.5	[31]
OIPL	Zn//NTP@Cu	2	~ 99	1400	0.56	[32]
ZA	Zn_3Hg//Ti	5	99.98	1000	2.5	[33]
ZA	$Zn_{0.73}Al_{0.27}$//Ti	5	99.13	480	0.48	[34]
ZA	Zn-Cu//Cu	5	~ 99	200	0.1	[35]
ZA	Zn-Mn//Cu	10	99.6	2500	2.5	[36]
ZA	Zn//Sn@Cu	1	98.88	210	0.21	[37]
ZA	Zn//ZnSe@Ti	2	99.2	400	0.2	[38]
ZA	PCu@Zn//Cu	5	97.55	450	0.45	[39]
**ZA**	**Zn-Ti//Cu**	**5**	**99.85**	**4000**	**4**	**This work**

Supplementary Table 3. Comparison of cyclic performance with other lab-level pouch cells using vanadium-based cathodes. Note: M stands for multi-layer, while S represents single-layer.

Type	Cell configuration	Current density ($A\ g^{-1}$)	Single-layer areal capacity ($mAh\ cm^{-2}$)	Cycle number	Capacity retention (%)	Ref.
Separator	CP-GF//$NH_4V_4O_{10}$ (S)	1	0.394	450	85	40
Separator	Janus separator//$NH_4V_4O_{10}$ (S)	2	0.1869	260	87.4	41
Electrolyte	Zeolite-modified//VO_2 (S)	/	~ 0.84	100	89.7	42
Electrolyte	0.02 M $BMIm^+$//$NH_4V_4O_{10}$ (S)	0.4	1.33	240	77.5	43
Electrolyte	HEE-1.2-3//$NH_4V_4O_{10}$ (S)	0.5	0.5	500	87	44
Electrolyte	Quasi-solid//$Na_xV_2O_5\cdot nH_2O$ (S)	0.1	0.25	50	62.6	45
Electrolyte	PCZ-gel//$NH_4V_4O_{10}$ (S)	1	~ 0.85	150	84	46
Electrolyte	SPS10//$V_2O_5\cdot H_2O$ (S)	0.14	1.66	200	82.8	47
Electrolyte	ImS/ZSO//$NaV_3O_8\cdot 1.5H_2O$ (M)	1	~ 0.99	115	91.4	48
Electrolyte	67Malt/ZS//$NH_4V_4O_{10}$ (S)	0.05	~ 0.37	200	93.7	49
Electrolyte	Sulfolane-H_2O//$V_2O_5\cdot nH_2O$ (M)	0.5	~ 1.1	55	81.6	50
Electrolyte	P20//$NaV_3O_8\cdot 1.5H_2O$ (S)	0.2	~ 0.74	50	105	51
Anode	3D Ni-Zn//PANI-$V_2O_5\cdot nH_2O$ (S)	10	~ 0.32	1000	80	52
Anode	UiO-66-$(COOH)_2$@Zn// $V_2O_5\cdot H_2O$ (S)	1	~ 0.3	1000	85	53
Anode	Cu-Zn@Zn//V_2O_5 (S)	2	0.234	600	88.2	54
Anode	NTP@Zn//$NaV_3O_8\cdot 1.5H_2O$ (S)	2	~ 0.83	250	76	32
Anode	SIP@Zn//$Mg_{0.1}V_2O_5\cdot H_2O$ (S)	5	~ 0.33	500	61	30
Anode	ZCO@Zn//$NH_4V_4O_{10}$ (S)	2	0.3	60	55.4	24
Anode	ZP@Zn//V_6O_{13} (S)	1	0.3266	100	86.1	19
**Anode**	**Zn-Ti//$NH_4V_4O_{10}$ (M)**	**1**	**1.42**	**500**	**85**	**This work**

Supplementary Table 4. The physical parameters for simulation.

Parameter	Expression	Value	Description
Cinit	3000[mol m^{-3}]	3000 mol m^{-3}	Initial concentration
T0	298.15[K]	298.15 K	System temperature
i0_ref	15[A m^{-2}]	15 A m^{-2}	Exchange current density
phis_anode	0.02[V]	0.02 V	Anode potential
phis_cathode	−0.02[V]	−0.02 V	Cathode potential
alpha_c	0.5[1]	0.5	Symmetry factor
alpha_a	1.5[1]	1.5	Symmetry factor
z_net	2[1]	2	Net species charge
z_Zn	z_net[1]	2	Charge, species Zn
z_SO4	−z_net[1]	−2	Charge, species SO4
D_Zn	1e−9[m^2 s^{-1}]	1E−9 m^2 s^{-1}	Diffusivity, species Zn
D_SO4	D_Zn	1E−9 m^2 s^{-1}	Diffusivity, species SO4
MZn	0.06538[kg mol^{-1}]	0.06538 kg mol^{-1}	Molar mass of zinc
rhoZn	7140[kg m^{-3}]	7140 kg m^{-3}	Density of zinc

Supplementary References

1. Ghosh, G., Delsante, S., Borzone, G., Asta, M. & Ferro, R. Phase stability and cohesive properties of Ti-Zn intermetallics: First-principles calculations and experimental results. *Acta Mater.* **54**, 4977-4997 (2006).
2. Zhao, R. et al. Prioritizing hetero-metallic interfaces via thermodynamics inertia and kinetics zincophilia metrics for tough Zn-based aqueous batteries. *Adv. Mater.* **35**, 2209288 (2023).
3. Xie, X. et al. Manipulating the ion-transfer kinetics and interface stability for high-performance zinc metal anodes. *Energy Environ. Sci.* **13**, 503-510 (2020).
4. Wang, Y. et al. Sulfolane-containing aqueous electrolyte solutions for producing efficient ampere-hour-level zinc metal battery pouch cells. *Nat. Commun.* **14**, 1828 (2023).
5. Hao, J., Yuan, L., Zhu, Y., Jaroniec, M. & Qiao, S. Triple-function electrolyte regulation toward advanced aqueous Zn-ion batteries. *Adv. Mater.* **34**, 2206963 (2022).
6. Li, S. et al. Design and synthesis of a π-conjugated N-heteroaromatic material for aqueous zinc-organic batteries with ultrahigh rate and extremely long life. *Adv. Mater.*, 2207115 (2023).
7. Li, Q. et al. Tailoring the metal electrode morphology via electrochemical protocol optimization for long-lasting aqueous zinc batteries. *Nat. Commun.* **13**, 3699 (2022).
8. Seo, J. et al. Electrodeposition-guided pre-passivation of Li-metal anode to enable long stable cycling of practical Li-metal batteries. *Energy Stor. Mater.* **60**, 102827 (2023).
9. Pei, A., Zheng, G., Shi, F., Li, Y. & Cui, Y. Nanoscale nucleation and growth of electrodeposited lithium metal. *Nano Lett.* **17**, 1132-1139 (2017).
10. Sagane, F. et al. Effects of current densities on the lithium plating morphology at a lithium phosphorus oxynitride glass electrolyte/copper thin film interface. *J. Power Sources* **233**, 34-42 (2013).
11. Ely, D. R. & García, R. E. Heterogeneous nucleation and growth of lithium electrodeposits on negative electrodes. *J. Electrochem. Soc.* **160**, A662-A668 (2013).
12. Wan, M. et al. Mechanical rolling formation of interpenetrated lithium metal/lithium tin alloy foil for ultrahigh-rate battery anode. *Nat. Commun.* **11**, 829 (2020).
13. Yao, Z. et al. Fast ion/electron conducting scaffold of Li-Zn dual-phase alloy enable uniform deposition of Li metal at high current densities. *J. Energy Chem.* **51**, 285-292 (2020).
14. Wang, D. et al. Insight on organic molecules in aqueous Zn-ion batteries with an emphasis on the Zn anode regulation. *Adv. Energy Mater.* **12**, 2102707 (2022).
15. Kundu, D. et al. Aqueous vs. nonaqueous Zn-ion batteries: Consequences of the desolvation penalty at the interface. *Energy Environ. Sci.* **11**, 881-892 (2018).

16. Cao, L. et al. Fluorinated interphase enables reversible aqueous zinc battery chemistries. *Nat. Nanotechnol.* **16**, 902-910 (2021).
17. Zhou, J. et al. Ultrathin surface coating of nitrogen-doped graphene enables stable zinc anodes for aqueous zinc-ion batteries. *Adv. Mater.* **33**, 2101649 (2021).
18. Ma, C. et al. Recyclable and ultrafast fabrication of zinc oxide interface layer enabling highly reversible dendrite-free Zn anode. *ACS Energy Lett.* **8**, 1201-1208 (2023).
19. Xing, Z. et al. Zincophilic electrode interphase with appended proton reservoir ability stabilizes Zn metal anodes. *Angew. Chem. Int. Ed.* **62**, e202215324 (2023).
20. Ma, L. et al. Toward practical high-areal-capacity aqueous zinc-metal batteries: Quantifying hydrogen evolution and a solid-ion conductor for stable zinc anodes. *Adv. Mater.* **33**, 2007406 (2021).
21. Peng, H. et al. Constructing fast-ion-conductive disordered interphase for high-performance zinc-ion and zinc-iodine batteries. *Matter* **5**, 4363-4378 (2022).
22. Liu, H. et al. Navigating fast and uniform zinc deposition via a versatile metal-organic complex interphase. *Energy Environ. Sci.* **15**, 1872-1881 (2022).
23. He, X. et al. Anion concentration gradient-assisted construction of a solid-electrolyte interphase for a stable zinc metal anode at high rates. *J. Am. Chem. Soc.* **144**, 11168-11177 (2022).
24. Wang, P. et al. Spontaneous construction of nucleophilic carbonyl-containing interphase toward ultrastable zinc-metal anodes. *Adv. Mater.* **34**, 2202733 (2022).
25. Liu, Q. et al. Elastomer-alginate interface for high-power and high-energy Zn metal anodes. *Adv. Energy Mater.* **12**, 2200318 (2022).
26. Chen, R. et al. Zwitterionic bifunctional layer for reversible Zn anode. *ACS Energy Lett.* **7**, 1719-1727 (2022).
27. Zhao, Z. et al. Horizontally arranged zinc platelet electrodeposits modulated by fluorinated covalent organic framework film for high-rate and durable aqueous zinc ion batteries. *Nat. Commun.* **12**, 6606 (2021).
28. Peng, H. et al. Intercalation of organics into layered structures enables superior interface compatibility and fast charge diffusion for dendrite-free Zn anodes. *Energy Environ. Sci.* **15**, 1682-1693 (2022).
29. Guo, Z. et al. A dynamic and self-adapting interface coating for stable Zn-metal anodes. *Adv. Mater.* **34**, 2105133 (2022).
30. Zhao, M. et al. Semi-immobilized ionic liquid regulator with fast kinetics toward highly stable zinc anode under −35 to 60 °C. *Adv. Mater.* **34**, 2203153 (2022).

31. Guo, C. et al. Synergistic manipulation of hydrogen evolution and zinc ion flux in metal-covalent organic frameworks for dendrite-free Zn-based aqueous batteries. *Angew. Chem. Int. Ed.* **61**, e202210871 (2022).
32. Zhao, M. et al. Simultaneously stabilizing both electrodes and electrolytes by a self-separating organometallics interface for high-performance zinc-ion batteries at wide temperatures. *Adv. Mater.* **34**, 2206239 (2022).
33. Tao, H., Hou, Z., Zhang, L., Yang, X. & Fan, L. Manipulating alloying reaction to achieve the stable and dendrite-free zinc metal anodes. *Chem. Eng. J.* **450**, 138048 (2022).
34. Zheng, J. et al. Electrostatic shielding regulation of magnetron sputtered Al-based alloy protective coatings enables highly reversible zinc anodes. *Nano Lett.* **22**, 1017-1023 (2022).
35. Tian, H. et al. Three-dimensional Zn-based alloys for dendrite-free aqueous Zn battery in dual-cation electrolytes. *Nat. Commun.* **13**, 7922 (2022).
36. Tian, H. et al. Stable, high-performance, dendrite-free, seawater-based aqueous batteries. *Nat. Commun.* **12**, 237 (2021).
37. Li, S. et al. Toward planar and dendrite-free Zn electrodepositions by regulating Sn-crystal textured surface. *Adv. Mater.* **33**, 2008424 (2021).
38. Yang, X. et al. Interfacial manipulation via in situ grown ZnSe cultivator toward highly reversible Zn metal anodes. *Adv. Mater.* **33**, 2105951 (2021).
39. Zhou, J. et al. Establishing thermal infusion method for stable zinc metal anodes in aqueous zinc-ion batteries. *Adv. Mater.* **34**, 2200782 (2022).
40. Yang, Z. et al. A piece of common cellulose paper but with outstanding functions for advanced aqueous zinc-ion batteries. *Mater. Today Energy* **28**, 101076 (2022).
41. Zhang, X. et al. An ion-sieving Janus separator toward planar electrodeposition for deeply rechargeable Zn-metal anodes. *Adv. Mater.* **34**, 2205175 (2022).
42. Yang, H. et al. Reducing water activity by zeolite molecular sieve membrane for long-life rechargeable zinc battery. *Adv. Mater.* **33**, 2102415 (2021).
43. Zhang, H. et al. Inducing the preferential growth of Zn (002) plane for long cycle aqueous Zn-ion batteries. *Adv. Energy Mater.* **13**, 2203254 (2023).
44. Han, M. et al. Hydrated eutectic electrolyte with ligand-oriented solvation shell to boost the stability of zinc battery. *Adv. Funct. Mater.* **32**, 2110957 (2022).
45. Guo, S. et al. Quasi-solid electrolyte design and in situ construction of dual electrolyte/electrode interphases for high-stability zinc metal battery. *Adv. Energy Mater.* **12**, 2200730 (2022).

46. Zhang, H., Gan, X., Song, Z. & Zhou, J. Amphoteric cellulose-based double-network hydrogel electrolyte toward ultra-stable Zn anode. *Angew. Chem. Int. Ed.* **62**, e202217833 (2023).
47. Lin, Y. et al. Dendrite-free Zn anode enabled by anionic surfactant-induced horizontal growth for highly-stable aqueous Zn-ion pouch cells. *Energy Environ. Sci.* **16**, 687-697 (2023).
48. Lv, Y. et al. Engineering a self-adaptive electric double layer on both electrodes for high-performance zinc metal batteries. *Energy Environ. Sci.* **15**, 4748-4760 (2022).
49. Chen, W. et al. Hydrogen bond-functionalized massive solvation modules stabilizing bilateral interfaces. *Adv. Funct. Mater.* **32**, 2112609 (2022).
50. Li, M. et al. Comprehensive H_2O molecules regulation via deep eutectic solvents for ultra-stable zinc metal anode. *Angew. Chem. Int. Ed.* **62**, e202215552 (2023).
51. Liu, D. et al. Regulating the electrolyte solvation structure enables ultralong lifespan vanadium-based cathodes with excellent low-temperature performance. *Adv. Funct. Mater.* **32**, 2111714 (2022).
52. Zhang, G. et al. 3D-printed multi-channel metal lattices enabling localized electric-field redistribution for dendrite-free aqueous Zn ion batteries. *Adv. Energy Mater.* **11**, 2003927 (2021).
53. Xin, W. et al. Metal-organic frameworks with carboxyl functionalized channels as multifunctional ion-conductive interphase for highly reversible Zn anode. *Energy Stor. Mater.* **56**, 76-86 (2023).
54. Li, B. et al. Multicomponent copper-zinc alloy layer enabling ultra-stable zinc metal anode of aqueous Zn-ion battery. *Angew. Chem. Int. Ed.* **61**, e202212587 (2022).
