# Supplementary material for: Tailoring grain boundary stability of zinc-titanium alloy for long-lasting aqueous zinc batteries
Source: Nat Commun. 2023 Nov 4;14:7080. doi: 10.1038/s41467-023-42919-7 (PMC10625522; doi:10.1038/s41467-023-42919-7)
Supplement: Supplementary file 3 — Reporting Summary [file 41467_2023_42919_MOESM3_ESM.pdf]

nature portfolio

Corresponding author(s): Jiang Zhou
(reference number: NCOMMS-23-18869B)

Last updated by author(s): Oct 25, 2023

Reporting Summary

Nature Portfolio wishes to improve the reproducibility of the work that we publish. This form provides structure for consistency and transparency in reporting. For further information on Nature Portfolio policies, see our Editorial Policies and the Editorial Policy Checklist.

Statistics

For all statistical analyses, confirm that the following items are present in the figure legend, table legend, main text, or Methods section.

n/a	Confirmed	
☐	☒	The exact sample size (*n*) for each experimental group/condition, given as a discrete number and unit of measurement
☐	☒	A statement on whether measurements were taken from distinct samples or whether the same sample was measured repeatedly
☒	☐	The statistical test(s) used AND whether they are one- or two-sided<br>*Only common tests should be described solely by name; describe more complex techniques in the Methods section.*
☒	☐	A description of all covariates tested
☒	☐	A description of any assumptions or corrections, such as tests of normality and adjustment for multiple comparisons
☐	☒	A full description of the statistical parameters including central tendency (e.g. means) or other basic estimates (e.g. regression coefficient) AND variation (e.g. standard deviation) or associated estimates of uncertainty (e.g. confidence intervals)
☒	☐	For null hypothesis testing, the test statistic (e.g. *F*, *t*, *r*) with confidence intervals, effect sizes, degrees of freedom and *P* value noted<br>*Give P values as exact values whenever suitable.*
☒	☐	For Bayesian analysis, information on the choice of priors and Markov chain Monte Carlo settings
☒	☐	For hierarchical and complex designs, identification of the appropriate level for tests and full reporting of outcomes
☒	☐	Estimates of effect sizes (e.g. Cohen's *d*, Pearson's *r*), indicating how they were calculated

Our web collection on statistics for biologists contains articles on many of the points above.

Software and code

Policy information about availability of computer code

Data collection	The electrochemical data were collected by the "LAND CT2001A", "Neware CT-4008T", "Multi Autolab/M204" and "CHI660E" system. Crystallographic phase and chemical composition data were investigated by the "Rigaku D/max2500" and "Spectro Blue Sop" system. Surface topography and microstructure were characterized by the "TESCAN MIRA4 LMH", "Bruker Dimension Icon", "KEYENCE VK-X1000", "NordlysMax2" and "FEI Tecani F30" system. Operando visualization was carried out using the "CEWEI LW750LJT" and "BJSCISTAR" system.
Data analysis	The electrochemical data were analyzed by the "LANDdt V7.4", "BTSDA 8.1.0.6", "Nova 2.1.5" and "CHI660E", respectively. Crystallographic phase and chemical composition data were processed using the "MDI Jade 9" and "Smart Analyzer Vision". Surface topography and microstructure were investigated by the "Essence", "NanoScope Analysis 1.9", "MultiFileAnalyzer", "Channel 5" and "DigitalMicrograph GMS3". Operando visualization was analyzed by the "Capture2.1" software. "ImageJ" was employed for statistical analysis. Density functional theory calculations were performed by the "Vienna Ab-initio Simulation Package 5.4.4". The finite element simulations were conducted using the "COMSOL Multiphysics 5.6". "OriginPro 2022 SR1", "Cinema 4D" and "Microsoft PowerPoint 2019" were utilized for creating and integrating the graphs.

For manuscripts utilizing custom algorithms or software that are central to the research but not yet described in published literature, software must be made available to editors and reviewers. We strongly encourage code deposition in a community repository (e.g. GitHub). See the Nature Portfolio guidelines for submitting code & software for further information.

Data

Policy information about availability of data

All manuscripts must include a data availability statement. This statement should provide the following information, where applicable:

- Accession codes, unique identifiers, or web links for publicly available datasets
- A description of any restrictions on data availability
- For clinical datasets or third party data, please ensure that the statement adheres to our policy

All data that support the findings of this study are presented in the Manuscript and Supplementary Information, or are available from the corresponding author upon reasonable request. Source data are provided with this paper.

Research involving human participants, their data, or biological material

Policy information about studies with human participants or human data. See also policy information about sex, gender (identity/presentation), and sexual orientation and race, ethnicity and racism.

Reporting on sex and gender	No research involving sex and gender in our paper. There is no information about the sex, gender and sexual orientation in this research.
Reporting on race, ethnicity, or other socially relevant groupings	No research involving socially relevant groupings in our paper. There is no information about the confounding variables in our analyses.
Population characteristics	No research involving human research in our paper. There is no information about the covariate-relevant population characteristics of the human research participants.
Recruitment	No research involving how participants are recruited in our paper. There is no information about the potential self-selection bias or other biases that might exist, as well as their potential impact on the results.
Ethics oversight	No research involving human participants, sex, gender, sexual orientation, race, ethnicity and racism in our paper. There is no information about the organization(s) that approved the study protocol.

Note that full information on the approval of the study protocol must also be provided in the manuscript.

Field-specific reporting

Please select the one below that is the best fit for your research. If you are not sure, read the appropriate sections before making your selection.

☐ Life sciences ☐ Behavioural & social sciences ☒ Ecological, evolutionary & environmental sciences

For a reference copy of the document with all sections, see nature.com/documents/nr-reporting-summary-flat.pdf

Ecological, evolutionary & environmental sciences study design

All studies must disclose on these points even when the disclosure is negative.

Study description	We delve into the correlation between surface microstructure and corrosion behavior, and propose an innovative grain boundary engineering via alloying strategy to tailor the interfacial stability and deposition behavior of Zn anodes. The Ti-containing intermetallic compounds are formed and thermodynamically stabilized at grain boundaries independent of Zn migration, which substantially suppress the HER-induced intergranular corrosion. Moreover, the enhanced zincophilicity of Zn-Ti alloy results in a diminished nucleation energy barrier, facilitating favorable nucleation and growth mode and leading to homogeneous Zn deposition. Our discovery specifies a seldom-addressed perspective concerning microstructure features and may invoke a paradigm shift to future polycrystalline anode design.
Research sample	A Zn-Ti dual-phase alloy, in which Ti-containing intermetallic compounds are preferentially distributed along the grain boundaries, has been developed. These compounds can thermodynamically remain stable in mildly acidic electrolytes and exhibit a strong affinity for Zn, consequently tailoring the stability of Zn anodes and regulating the Zn deposition behavior.
Sampling strategy	The sample selection does not involve statistical methods. The electrode sample sizes (unless stated otherwise, 100 μm in thickness and 12 mm in diameter) are specified in the experimental section, representing a parameter commonly employed for coin cell testing.
Data collection	Y.Z., X.Z. and J.Z. conceived the idea and designed the experiments. S.L. and J.Z. provided critical guidance on the project. Y.Z., X.Z. and M.C. carried out the synthesis, materials characterizations and electrochemical measurements. Y.Z. performed the finite element simulations. S.G. conducted the theoretical calculations. B.L. provided important feedback that facilitated a systematic data analysis. Y.Z. and X.Z. wrote the manuscript. All authors contributed to the discussion of the results.
Timing and spatial scale	2022-2023. Data collection commences from the initial stage of electrochemical testing and concludes upon the completion of the testing process.

Data exclusions	No data were excluded.
Reproducibility	The experiments were replicated over multiple days to ensure consistent results. All attempts to repeat the experiments were successful.
Randomization	This study does not involve quantitative methods that would require randomization.
Blinding	Our study (field: electrochemistry) does not include experiments that would require blinding.

Did the study involve field work? ☐ Yes ☒ No

Reporting for specific materials, systems and methods

We require information from authors about some types of materials, experimental systems and methods used in many studies. Here, indicate whether each material, system or method listed is relevant to your study. If you are not sure if a list item applies to your research, read the appropriate section before selecting a response.

Materials & experimental systems

n/a	Involved in the study
☒	☐ Antibodies
☒	☐ Eukaryotic cell lines
☒	☐ Palaeontology and archaeology
☒	☐ Animals and other organisms
☒	☐ Clinical data
☒	☐ Dual use research of concern
☒	☐ Plants

Methods

n/a	Involved in the study
☒	☐ ChIP-seq
☒	☐ Flow cytometry
☒	☐ MRI-based neuroimaging
